# Assessing the relationship between gut microbiota and hyperprolactinemia: A bidirectional two-sample Mendelian randomization study

**DOI:** 10.1097/MD.0000000000045484

**Published:** 2025-10-24

**Authors:** Teng Qi, Yujie Hu, Xiaorui Zhou, Lanhui Zhang, Xulong Zhu, Mingkun Zhang, Zhenping Ouyang, Miao Wei, Sujin Wang, Chuanlin Zhao, Fuqing Ji

**Affiliations:** a School of Medicine, The Chinese University of Hong Kong, Shenzhen, China; b Key Laboratory of Resource Biology and Biotechnology in Western China, Ministry of Education, School of Medicine, Northwest University, Xi’an, Shaanxi Province, P.R. China; c School of Medicine, Tianjin University, Tianjin, P.R.China; d Department of Traditional Chinese Medicine, Xi’an NO.3 Hospital, the Affiliated Hospital of Northwest University, Xi’an, Shaanxi Province, P.R. China; e Department of Surgical Oncology, Shaanxi Provincial People’s Hospital, Xi`an, P.R. China; f Department of Thyroid Breast and Vascular Surgery, Xijing Hospital, The Fourth Military Medical University, Xi’an, P.R. China.

**Keywords:** genetic epidemiology, gut microbiota, hyperprolactinemia, Mendelian randomization

## Abstract

The relationship between hyperprolactinemia and gut microbiota remains unclear at present. This study employs a Mendelian randomization (MR) approach to assess the potential causal links between gut microbiota and the incidence of hyperprolactinemia. Genetic instrumental variables associated with gut microbiota were identified through a genome-wide association study involving 18,340 participants. Summary statistics regarding hyperprolactinemia were obtained from FinnGen R10, comprising 1099 cases and 395,289 controls. The primary analysis utilized the inverse-variance weighted method. Additionally, we employed the weighted-median method, MR-Egger regression, and MR pleiotropy residual sum and outlier test to validate the robustness of our findings. Subsequently, a reverse MR analysis was conducted to assess the potential for reverse causation. We identified suggestive associations between 7 bacterial traits and the risk of hyperprolactinemia (odds ratio [OR]: 0.685; 95% confidence interval [CI]: 0.483 to 0.97; *P* = .033 for Family *Bacteroidales S24.7*; OR: 1.589; 95% CI: 1.057 to 2.389; *P* = .026 for Genus *Ruminococcus gauvreauii group*; OR: 0.686; 95% CI: 0.522 to 0.901; *P* = .007 for Genus *Anaerofilumgroup*; OR: 1.333; 95% CI: 1.017 to 1.747; *P* = .037 for Genus *Eisenbergiella group*; OR: 0.595; 95% CI: 0.416 to 0.852; *P* = .005 for Genus *Erysipelotrichaceae UCG003 group*; OR: 1.3986; 95% CI: 1.00 to 1.954; *P* = .005 for Genus *Ruminococcaceae UCG014 group* and OR: 0.781; 95% CI: 0.612 to 0.998; *P* = .048 for Genus *Peptococcus group*).We did not find statistically significant associations between hyperprolactinemia and these 7 bacterial traits in the reverse MR analysis. Our systematic analysis provides evidence supporting a potential causal relationship between specific gut microbiota taxa and the risk of hyperprolactinemia.

## 1. Introduction

Hyperprolactinemia is a prevalent endocrine disorder of the hypothalamic-pituitary axis, with prolactinoma being one of its clinical causes.^[1]^ In a recent epidemiological survey of hyperprolactinemic patients in Saudi, the majority of cases were found to occur in women (86.8%) and were more frequently observed in patients aged 21 to 40 years.^[2]^ Typical symptoms of hyperprolactinemia include hypogonadism, infertility, galactorrhea, osteopenia, and tumor mass effects.^[1]^ Patients with this condition may experience a significant reduction in their quality of life due to severe breast symptoms, breastfeeding discomfort, menstrual changes, and even male erectile dysfunction.^[3]^ Moreover, hyperprolactinemia has been reported to be associated with endometriosis in infertile women.^[4]^ Currently, dopamine agonists and surgery are the treatments for hyperprolactinemia.^[5]^ However, due to the complex nature of this endocrine disease, it is imperative to identify its pathogenesis and develop more effective and minimally invasive treatment options.

The pathophysiological mechanism of hyperprolactinemia is multifactorial; however, the current understanding of its underlying causes remains incomplete. Studies have shown that there is a strong genetic component in the risk of hyperprolactinemia.^[6]^The genetic mechanism of hyperprolactinemia may not be a simple single gene mutation, but multiple gene changes, such as prolactin receptor mutations and polymorphisms.^[7]^ Gut microbiota may influence the occurrence and progression of diseases by producing certain metabolites and regulating disease-related genes, thereby causing changes in endocrine system signaling pathways.^[8]^

Recent evidence suggests that the pathogenesis of hyperprolactinemia may be closely related to the gut microbiota.^[9]^ In an animal study, the researchers found that severe hyperprolactinemia can exacerbate metabolic imbalances induced by a high-fat diet,^[10]^which may be related to changes in the gut microbiota.^[11]^Therefore, gut microbes may directly or indirectly influence the occurrence and development of hyperprolactinemia. To date, there have been no studies establishing a link between the gut microbiota and hyperprolactinemia. Therefore, it remains unclear whether a causal relationship exists.

Mendelian randomization is an established epidemiological method that combines observational data to directly estimate causal effects.^[12]^ It has become a valuable tool for analyzing potential causal relationships between the gut microbiota and disease risk genes.^[13–17]^ In order to investigate the potential causal relationship between the gut microbiota and hyperprolactinemia, as well as identify specific classifications of pathogenic bacteria, we conducted a Mendelian randomization (MR) Analysis using pooled data from genome-wide association studies.

## 2. Materials and methods

### 2.1. Outcome data sources

**Figure 1.**
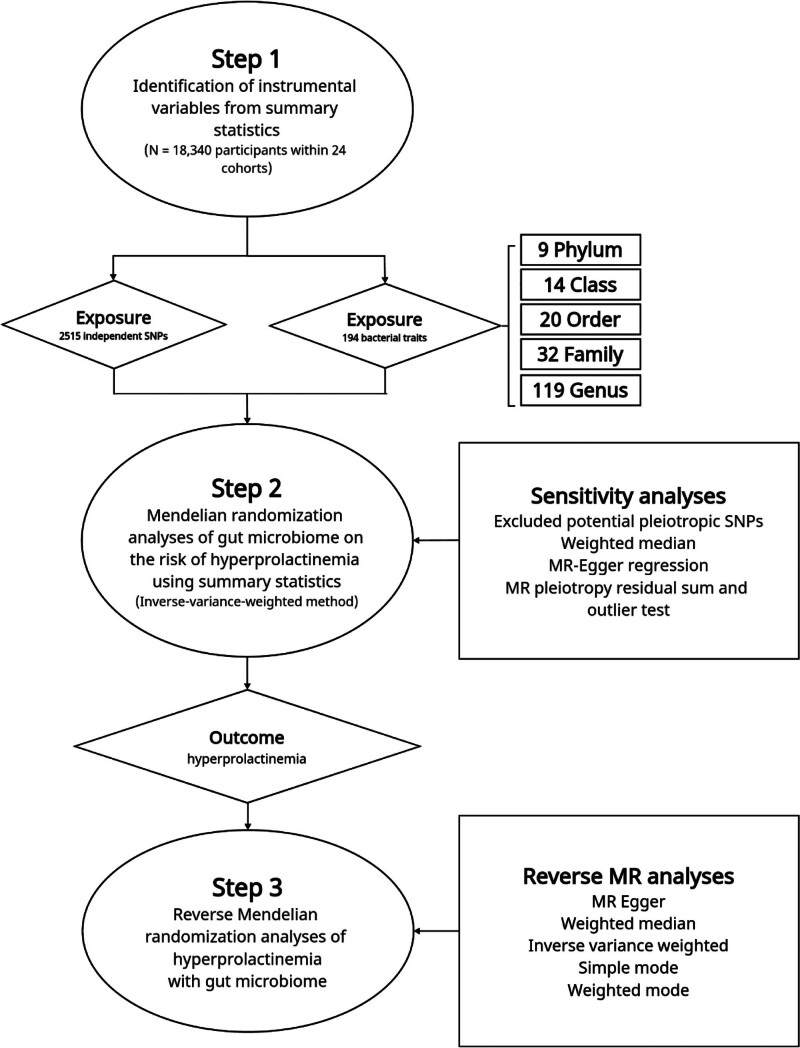
The study design of the associations of gut microbiota and hyperprolactinemia. MR = Mendelian randomization, SNP = single-nucleotide polymorphism.

The overall process design of this study is shown in Figure 1. We obtained summary statistics for human gut microbiome and abdominal aortic aneurysm from a recent meta-analysis of genome-wide association studies (GWAS).^[18]^ The meta-analysis of human gut microbiome included 18,340 participants from 24 different cohorts. More information about this study can be found elsewhere.^[18]^ In summary, the study combined data from various countries, such as United States of America, Canada, Germany, Denmark, Netherlands, Belgium, Sweden, Finland, and the United Kingdom. It integrated 16S rRNA gene sequencing profiles with genotyping data and performed association analyses while adjusting for factors such as age, sex, technical covariates, and genetic principal components.^[18]^ The data for hyperprolactinemia were derived from samples of Finland ancestry, including 1099 disease samples and 395,289 control samples.^[19]^ Since our study utilized publicly available summary data, there was no need for additional ethics approval or participant consent. The detailed information of the data sources used in this MR study can be found in Table 1.

**Table 1:** Details of the genome-wide association studies and datasets used in our analyses.

Exposure or outcome	Sample size	Ancestry	Links for data download
Human gut microbiome	18,340 participants	Mixed	https://mibiogen.gcc.rug.nl
hyperprolactinemia	1099 cases, 395,289 controls	Finland	https://risteys.finregistry.fi/endpoints/E4_HYPERPRO

### 2.2. Selection of instrumental variables

Initially, we excluded 15 unidentified bacterial traits from our analysis, leaving us with a remaining set of 194 bacterial traits that span 9 phyla, 14 classes, 20 orders, 32 families, and 119 genera. Subsequently, we applied a significance threshold of *P <* 1.0 × 10^−5^ to select instrumental variables (IVs). To ensure genetic independence between loci, we utilized a linkage disequilibrium (LD) threshold of *R*^*2*^* *< 0.001 and clumping distance = 10,000 kb using the “TwoSampleMR” package on the 1000 Genomes EUR data. For each associated trait, we retained single-nucleotide polymorphisms (SNPs) with the lowest *P*-value for subsequent clumping analysis alongside the other 194 bacterial traits. This process resulted in a total of 2515 independent SNPs that were found to be associated with these bacterial traits. In the reverse MR analysis for hyperprolactinemia, we used the threshold for *P* < 1 × 10^−5^, as previously described in Table 2.^[20,21]^ Extracting pertinent information such as the effect allele, effect size (including *β*-value), standard error, and *P*-value for each SNP allowed us to calculate the proportion of variation explained (*R*^*2*^) and *F*-statistics to evaluate instrument strength. These calculations followed the equation:

**Table 2:** Effect estimates of the associations of hyperprolactinemia with 9 types of gut microbiota in the reverse MR analyses.

Gut microbiome	Methods	N.SNPs	OR	95% CI	*P*-value
Family Acidaminococcaceae	MR Egger	8	0.97	0.83–1.14	.753
	Weighted median	8	1.02	0.96–1.08	.509
	Inverse variance weighted	8	1.00	0.96–1.04	.966
	Simple mode	8	1.04	0.95–1.14	.387
	Weighted mode	8	1.03	0.95–1.14	.470
Family Bacteroidales S24.7 group	MR Egger	8	0.94	−0.25–0.12	.531
	Weighted median	8	0.96	−0.12–0.03	.285
	Inverse variance weighted	8	0.98	−0.08–0.03	.428
	Simple mode	8	0.97	−0.15–0.09	.655
	Weighted mode	8	0.96	−0.16–0.08	.536
Genus Eubacterium ruminantium group	MR Egger	8	1.02	−0.15–0.19	.806
	Weighted median	8	1.03	−0.04–0.10	.437
	Inverse variance weighted	8	1.05	−0.01–0.10	.103
	Simple mode	8	1.01	−0.09–0.11	.854
	Weighted mode	8	1.02	−0.09–0.12	.768
Genus Ruminococcus gauvreauii group	MR Egger	8	0.93	−0.21–0.05	.296
	Weighted median	8	1.01	−0.04–0.07	.638
	Inverse variance weighted	8	1.01	−0.03–0.05	.753
	Simple mode	8	1.02	−0.06–0.10	.621
	Weighted mode	8	1.02	−0.06–0.09	.648
Genus Anaerofilum	MR Egger	8	0.90	−0.35–0.14	.424
	Weighted median	8	0.99	−0.10–0.08	.798
	Inverse variance weighted	8	1.01	−0.06–0.09	.727
	Simple mode	8	0.98	−0.15–0.11	.723
	Weighted mode	8	0.98	−0.14–0.10	.754
Genus Eisenbergiella	MR Egger	8	0.90	−0.34–0.14	.437
	Weighted median	8	0.95	−0.14–0.04	.242
	Inverse variance weighted	8	0.97	−0.10–0.05	.473
	Simple mode	8	0.93	−0.20–0.06	.309
	Weighted mode	8	0.94	−0.19–0.06	.357
Genus Erysipelotrichaceae UCG003	MR Egger	8	1.09	−0.05–0.22	.249
	Weighted median	8	0.98	−0.08–0.04	.496
	Inverse variance weighted	8	1.00	−0.05–0.04	.850
	Simple mode	8	0.97	−0.12–0.06	.565
	Weighted mode	8	0.98	−0.12–0.07	.622
Genus Peptococcus	MR Egger	8	1.09	−0.13–0.31	.448
	Weighted median	8	1.04	−0.05–0.13	.382
	Inverse variance weighted	8	1.05	−0.01–0.11	.126
	Simple mode	8	0.99	−0.15–0.12	.869
	Weighted mode	8	1.03	−0.09–0.15	.649
Genus Ruminococcaceae UCG014	MR Egger	8	0.96	−0.18–0.10	.627
	Weighted median	8	1.00	−0.06–0.05	.877
	Inverse variance weighted	8	1.00	−0.04–0.10	.852
	Simple mode	8	1.02	−0.07–0.10	.751
	Weighted mode	8	0.98	−0.10–0.07	.722

$$\begin{array}{l} R^{2}=2\times MAF\times (1-MAF)\times \beta^{2}, \end{array}$$ (1)

$$\begin{array}{l} F=R^{2}\left(n-k-1\right)/k\left(1-R^{2}\right) \end{array}$$ (2)

where “MAF” represents the minor allele frequency of IVs used, “n” denotes the sample size, and “k” signifies the number of IVs employed.^[20,21]^

### 2.3. Statistical analysis

We employed various methods to evaluate the potential causal links between the gut microbiota and hyperprolactinemia. These approaches consisted of the fixed/random-effects inverse-variance weighted (IVW) method, weighted-median method, MR-Egger regression, and MR pleiotropy residual sum and outlier (MR-PRESSO) test. The IVW method was our primary analysis due to its accurate effect estimates and widespread usage in MR analysis.^[22–24]^ Initially, individual SNPs were utilized with the Wald estimator and Delta method to calculate ratio estimates. These estimates were then combined to obtain the main causal estimate.^[25]^ Cochran’s *Q* test was used to assess heterogeneity among selected SNPs. If heterogeneity was detected (*P < *.05), we applied the random-effects IVW method; otherwise, we utilized the fixed-effects IVW method.^[26]^

To ensure robust associations considering valid instruments and potential pleiotropic effects, sensitivity analysis were conducted. First, we implemented the weighted median method, which provided reliable causal effect estimates even when valid instruments are limited.^[27]^ This approach yields valid results even if <50% of information is derived from invalid instruments.^[27]^ Second, we performed MR-Egger regression to examine potential horizontal pleiotropy by evaluating the *P*-value of the intercept (<.05 indicates possible horizontal pleiotropy of SNPs).^[28]^ Last, we employed the MR-PRESSO test to identify outliers among SNPs through a global test of heterogeneity. After identifying and removing potential outliers, a corrected association result was obtained.^[29]^

To further explore potential directional pleiotropy, each SNP used as IVs was assessed for their associations with secondary phenotypes using GWAS Catalog (http://www.ebi.ac.uk/gwas, last accessed on August 23, 2023). Subsequently, MR analysis weas repeated after excluding SNPs associated with other phenotypes.

The associations between human gut microbiota and the risk of hyperprolactinemia were reported as odds ratios (ORs) with 95% confidence intervals (CIs). The reverse MR analysis was carried out only when there was unanimous support from all MR methods regarding the link between the gut microbiota and hyperprolactinemia. All MR analysis were conducted using R version 4.2.2 (https://www.r-project.org/) with the “Mendelian Randomization,” “TwoSampleMR,” and “MR-PRESSO” R packages.

## 3. Results

### 3.1. Main results of the 194 bacterial traits with the risk of hyperprolactinemia

**Figure 2.**
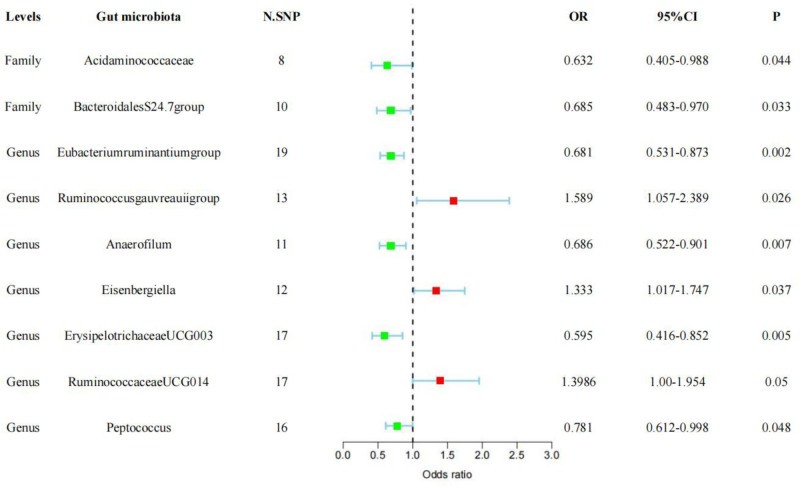
Forest plot of the associations between 9 genetically determined bacterial traits and the risk of hyperprolactinemia. CI = confidence interval, OR = odds ratio, SNP = single-nucleotide polymorphism.

Simply put, we observed suggestive evidence that 9 bacterial traits were associated with the risk of hyperprolactinemia using the IVW approach (Fig. 2). The MR results of the associations between all 194 bacterial traits and the risk of hyperprolactinemia are presented in Table S1, Supplemental Digital Content, https://links.lww.com/MD/Q466, and the IVs used for these 9 bacterial traits are listed in Table S2, Supplemental Digital Content, https://links.lww.com/MD/Q466.

**Figure 3.**
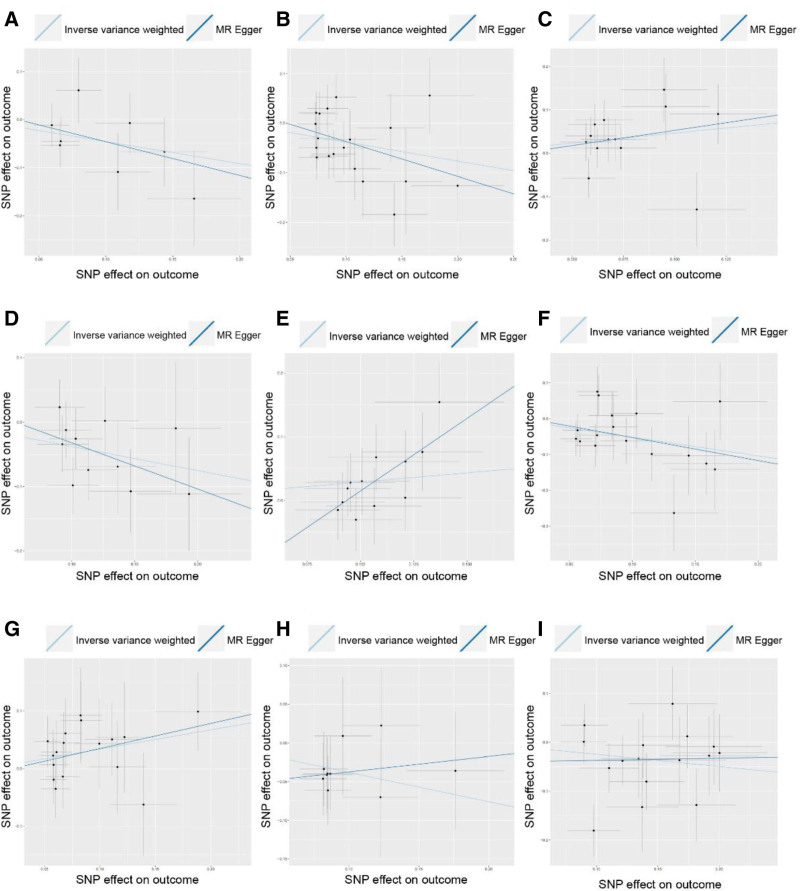
Scatter plot of the associations of genetic variants with 9 bacterial traits and the risk of hyperprolactinemia. (A) Family *Acidaminococcaceaeid*; (B) Genus *Eubacterium ruminantium group*; (C) Genus *Ruminococcus gauvreauii group*; (D) Genus *Anaerofilum*; (E) Genus *Eisenbergiella*; (F) Genus *Erysipelotrichaceae UCG003*; (G) Genus *Ruminococcaceae UCG014*; (H) Family *Bacteroidales S24.7 group*; (I) Genus *Peptococcus*. MR = Mendelian randomization, SNP = single-nucleotide polymorphism.

The IVW method and MR-Egger regression results revealed that certain bacterial groups, namely Family *Acidaminococcaceae* (Fig. 3A, OR: 0.632; 95% CI: 0.405 to 0.988; *P* = .044), Genus *Eubacterium ruminantium group* (Fig. 3B, OR: 0.681; 95% CI: 0.531 to 0.873; *P* = .002), Genus *Anaerofilum* (Fig. 3D, OR: 0.686; 95% CI: 0.522 to 0.901; *P* = .007), and Genus *Erysipelotrichaceae UCG003* (Fig. 3F, OR: 0.595; 95% CI: 0.416 to 0.852; *P* = .005), may serve as potential protective bacterial communities, exhibiting a negative association with hyperprolactinemia. Conversely, Genus *Ruminococcus gauvreaui group* (Fig. 3C, OR: 1.589; 95% CI: 1.057 to 2.389; *P* = .026), Genus *Eisenbergiella* (Fig. 3E, OR: 1.333; 95% CI: 1.017 to 1.747; *P* = .037), and Genus *Ruminococcaceae UCG014* (Fig. 3F, OR: 1.3986; 95% CI: 1.00 to 1.954; *P* = .05) may act as potential pathogenic bacterial communities, showing a positive association with hyperprolactinemia.

However, it should be noted that the impact of Family *Bacteroidales S24.7 group* (Fig. 3H, OR: 0.685; 95% CI: 0.483 to 0.970; *P* = .033) and Genus *Peptococcus* (Fig. 3I, OR: 0.781; 95% CI: 0.612 to 0.998; *P* = .048) on hyperprolactinemia appeared to be contradictory in the IVW analysis and MR-Egger regression. Further foundational research is needed to validate their influence on hyperprolactinemia.

**Figure 4.**
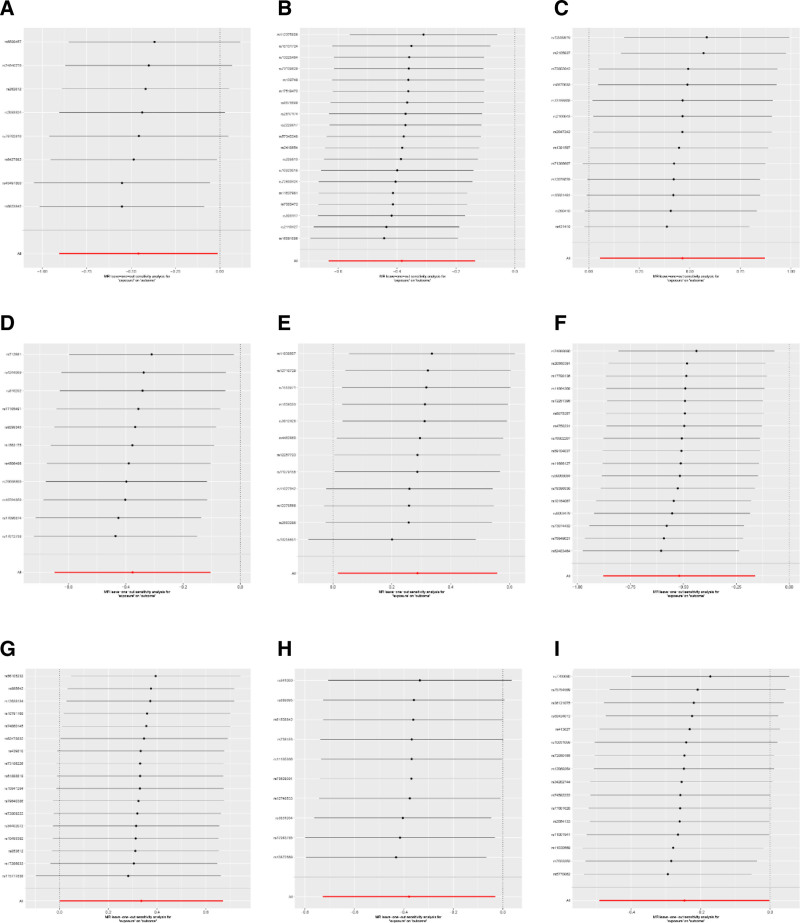
“Leave-one-out” sensitivity analysis for 9 types of gut microbiota on hyperprolactinemia. (A) Family *Acidaminococcaceaeid*; (B) Genus *Eubacterium ruminantium group*; (C) Genus *Ruminococcus gauvreauii group*; (D) Genus *Anaerofilum*; (E) Genus *Eisenbergiella*; (F) Genus *Erysipelotrichaceae UCG003*; (G) Genus *Ruminococcaceae UCG014*; (H) Family *Bacteroidales S24.7 group*; (I) Genus *Peptococcus*. MR = Mendelian randomization.

To further explore the impact of potential directional pleiotropy on the estimates of causal effects, we utilized the GWAS Catalog to identify single-nucleotide polymorphisms (SNPs) associated with the 9 bacterial traits under investigation. We found that 9 SNPs were associated with other traits, as shown in Table S3, Supplemental Digital Content, https://links.lww.com/MD/Q466. The leave-one-out method was employed to observe the minimal changes in results (Fig. 4), confirming the stability of the associations between various factors and the increased risk of hyperprolactinemia.

### 3.2. The results of reverse MR analysis

Subsequently, we conducted reverse MR analyses to evaluate the potential reverse associations between the 9 bacterial traits and hyperprolactinemia. We employed the IVW method for this purpose. However, we did not detect any statistically significant associations between hyperprolactinemia and any of these 9 bacterial traits. These findings remained consistent across sensitivity analysis, as presented in Table 2.

## 4. Discussion

The human gut microbiota exhibits a high sensitivity to pathophysiological changes within the body, making it crucial to investigate specific alterations in order to comprehend disease pathogenesis and enable accurate diagnoses.^[30]^ Such susceptibility to change can potentially lead to modifications in the microbiome, consequently contributing to the development of various diseases in humans.^[31]^ This 2-sample MR study identified 9 bacterial taxa that displayed potential associations with an increased prevalence of hyperprolactinemia. These taxa include the Family *Acidaminococcaceaeid* and *Bacteroidales S24.7 group*, as well as the genus *Ruminococcus gauvreauii group, Eisenbergiella, Ruminococcaceae UCG014, Eubacterium ruminantium group, Anaerofilum, Erysipelotrichaceae UCG003*, and *Peptococcus*. However, further sensitivity analysis utilizing different MR methods and restricted IV sets revealed that specifically, 7 bacterial taxa, *Ruminococcus gauvreauii group, Eisenbergiella, Ruminococcaceae UCG014, Acidaminococcaceaeid, Eubacterium ruminantium group, Anaerofilum*, and *Erysipelotrichaceae UCG003*, were associated with the risk of hyperprolactinemia.

In this study, a positive correlation was observed between hyperprolactinemia and 3 types of bacteria: Genus *Ruminococcus gauvreauii group*, Genus *Eisenbergiella*, and Genus *Ruminococcaceae UCG014*.^[32]^Genus *Ruminococcus gauvreauii group* in human disease-related research is less, only Djawad Radjabzadeh and others a cohort study on the gut microbiota of depression in a report. Previous studies have shown that hyperprolactinemia is closely related to depression.^[1,33]^ Patients with hyperprolactinemia usually have poor quality of life and personality characteristics such as anxiety and depression.^[34]^ So combining with the research of Genus *Ruminococcus gauvreauii group* relevant results suggest that Genus *Ruminococcus gauvreauii group* may through a series of pathophysiological mechanisms result in increased prolactin and cause depression. As a new microbiome found in human feces collected after bariatric surgery,^[35]^The Genus *Eisenbergiella* has been reported to be associated with multiple sclerosis, rheumatoid arthritis and high blood pressure.^[36–38]^ Therefore, we speculate that Genus *Eisenbergiella* may influence the occurrence and development of hyperprolactinemia through certain metabolites through inflammatory, lipid metabolism, and immune pathways.Genus *Ruminococcaceae UCG014* has been suggested to be associated with asthma in a previous MR study.^[39]^ Further inquiry into the gutMGene database revealed that metabolites of Genus *Ruminococcaceae UCG014* include indolepropionic acid, creatine, goandeoxycholic acid, 4-acetylaminobutyric acid, aquaposin, N(6) -methyllysine, n-acetyl-D-mannosamine, n-isovalerylglycine, etc. Its effect on hyperprolactinemia needs more in-depth basic research to clarify.^[40]^

After conducting our study, we discovered a negative correlation between hyperprolactinemia and the Family *Acidaminococcaceae*, Genus *eubacterium raminantium group*, Genus *Anaerofilum*, and Genus *Erysipelotrichaceae UCG003*. MR studies have linked Family *Acidaminococcaceae* to obstructive sleep apnea (OSA) disease severity and asthma.^[41]^ Further research has shown that these bacteria can ferment carbohydrates to produce metabolites such as butyric acid and propionic acid.^[42]^ It is conceivable that alterations in these metabolites by Family Acidaminococcaceae may modulate the body’s state, thereby contributing to the onset of diseases. The genus *Eubacterium ruminantium* has the capacity to generate short-chain fatty acids and bile metabolites, influencing the intestinal microenvironment. These metabolites play a crucial role in modulating the body’s inflammatory immune response, regulating blood glucose and cholesterol levels, and preserving the integrity of the intestinal barrier.^[43]^ Previous studies have confirmed that increased abundance of the Genus *Anaerofilum* has a positive effect on patients with depression.^[44]^ In conjunction with the findings of this study, the genus *Anaerofilum* could serve as a foundation for understanding the mechanistic link between depression and hyperprolactinemia.The association between *Erysipelotrichaceae UCG003* and cancer has been confirmed. The abundance of *Erysipelotrichaceae UCG003* is negatively correlated with glycerophospholipid metabolism which could be an essential mechanism for regulating metabolism and tumor development in vivo.^[45]^ Additionally,*Erysipelotrichaceae UCG003* is one of the main bacteria that produce butyrate, which affects intestinal flora and improves gastrointestinal function.^[45]^

In our research, we utilized genetic epidemiological methods to establish a causal relationship between the gut microbiota and hyperprolactinemia. Our IV analysis ensured that our results were less likely to be affected by weak instrument bias, as indicated by the *F*-statistic of our IVs exceeding the threshold of > 10. Furthermore, we conducted a reverse MR analysis to exclude the possibility of reverse causality. Moving forward, investigating the causal association between gut microbiota and disease development should be the focus of future research. Our MR analysis offers valuable insights into selecting specific gut bacteria for studying the role of gut microbiota in hyperprolactinemia pathogenesis.

Nevertheless, our study does have limitations. First, we only analyzed bacterial taxa at the genus level, rather than at a more detailed level, such as species or strain. Second, the majority of participants in our GWAS were of European descent, which may limit the generalizability of our findings to other ethnic groups. Third, we relaxed the threshold for selecting IVs for gut microbiota (*P* < 1.0 × 10^−5^) to obtain a sufficient number of IVs. Additionally, the effects of the bacterial traits we identified were relatively weak, and there were no other independent GWASs with an adequate sample size to validate our findings. Finally, due to the lack of specific information on the subtypes and gender of hyperprolactinemia, further research is needed when this information is available.

## 5. Conclusions

Our systematic analysis provides evidence supporting a potential causal relationship between specific gut microbiota taxa and the risk of hyperprolactinemia. Further research is needed to elucidate the mechanisms through which gut microbiota influence and expedite the development and progression of hyperprolactinemia.

## Author contributions

**Conceptualization:** Teng Qi, Fuqing Ji.

**Formal analysis:** Teng Qi, Zhenping Ouyang.

**Funding acquisition:** Fuqing Ji.

**Methodology:** Teng Qi, Lanhui Zhang.

**Software:** Teng Qi.

**Visualization:** Yujie Hu, Xiaorui Zhou, Sujin Wang.

**Writing – original draft:** Xiaorui Zhou, Miao Wei, Sujin Wang.

**Writing – review & editing:** Lanhui Zhang, Xulong Zhu, Mingkun Zhang, Chuanlin Zhao.

## Supplementary Material


