# Supplementary material for: Assessing the relationship between gut microbiota and hyperprolactinemia: A bidirectional two-sample Mendelian randomization study
Source: Medicine (Baltimore). 2025 Oct 24;104(43):e45484. doi: 10.1097/MD.0000000000045484 (PMC12558277; doi:10.1097/MD.0000000000045484)
Supplement: Supplementary file 1 [file medi-104-e45484-s001.docx]

**Supplementary Table 1** Effect estimates of the associations between 194 bacterial traits and the risk of hyperprolactinemia in MR analyses

| **Gut microbiota** | **Methods** | **N.SNP** | **b** | **se** | ***p-*value** |
| --- | --- | --- | --- | --- | --- |
| Class |  |  |  |  |  |
| Actinobacteria |  |  |  |  |  |
|  | MR Egger | 18 | -0.667 | 0.544 | 0.238 |
|  | Weighted median | 18 | 0.013 | 0.256 | 0.960 |
|  | Inverse variance weighted | 18 | 0.026 | 0.176 | 0.881 |
|  | Simple mode | 18 | 0.320 | 0.428 | 0.463 |
|  | Weighted mode | 18 | 0.349 | 0.360 | 0.345 |
| Alphaproteobacteria |  |  |  |  |  |
|  | MR Egger | 10 | -1.281 | 0.789 | 0.143 |
|  | Weighted median | 10 | 0.080 | 0.269 | 0.766 |
|  | Inverse variance weighted | 10 | 0.304 | 0.229 | 0.183 |
|  | Simple mode | 10 | 0.081 | 0.401 | 0.845 |
|  | Weighted mode | 10 | 0.088 | 0.358 | 0.811 |
| Bacilli |  |  |  |  |  |
|  | MR Egger | 22 | 0.306 | 0.444 | 0.498 |
|  | Weighted median | 22 | 0.003 | 0.229 | 0.990 |
|  | Inverse variance weighted | 22 | -0.129 | 0.169 | 0.444 |
|  | Simple mode | 22 | -0.466 | 0.463 | 0.326 |
|  | Weighted mode | 22 | 0.235 | 0.411 | 0.574 |
| Bacteroidia |  |  |  |  |  |
|  | MR Egger | 14 | 0.490 | 0.585 | 0.418 |
|  | Weighted median | 14 | 0.320 | 0.310 | 0.301 |
|  | Inverse variance weighted | 14 | 0.428 | 0.231 | 0.064 |
|  | Simple mode | 14 | -0.042 | 0.563 | 0.940 |
|  | Weighted mode | 14 | -0.024 | 0.507 | 0.962 |
| Clostridia |  |  |  |  |  |
|  | MR Egger | 16 | -0.762 | 0.890 | 0.406 |
|  | Weighted median | 16 | -0.345 | 0.290 | 0.234 |
|  | Inverse variance weighted | 16 | -0.260 | 0.215 | 0.226 |
|  | Simple mode | 16 | -0.597 | 0.460 | 0.213 |
|  | Weighted mode | 16 | -0.454 | 0.431 | 0.308 |
| Coriobacteriia |  |  |  |  |  |
|  | MR Egger | 17 | 0.409 | 0.485 | 0.411 |
|  | Weighted median | 17 | 0.345 | 0.271 | 0.202 |
|  | Inverse variance weighted | 17 | 0.300 | 0.193 | 0.121 |
|  | Simple mode | 17 | 0.302 | 0.430 | 0.494 |
|  | Weighted mode | 17 | 0.375 | 0.368 | 0.324 |
| Deltaproteobacteria |  |  |  |  |  |
|  | MR Egger | 14 | 0.044 | 0.548 | 0.937 |
|  | Weighted median | 14 | -0.107 | 0.284 | 0.708 |
|  | Inverse variance weighted | 14 | -0.224 | 0.202 | 0.268 |
|  | Simple mode | 14 | -0.136 | 0.423 | 0.752 |
|  | Weighted mode | 14 | -0.110 | 0.349 | 0.758 |
| Erysipelotrichia |  |  |  |  |  |
|  | MR Egger | 13 | -0.696 | 1.317 | 0.607 |
|  | Weighted median | 13 | -0.215 | 0.356 | 0,546 |
|  | Inverse variance weighted | 13 | -0.111 | 0.294 | 0.706 |
|  | Simple mode | 13 | -0.533 | 0.662 | 0.437 |
|  | Weighted mode | 13 | -0.560 | 0.604 | 0.372 |
| Lentisphaeria |  |  |  |  |  |
|  | MR Egger | 10 | 0.221 | 0.617 | 0.729 |
|  | Weighted median | 10 | 0.054 | 0.190 | 0.777 |
|  | Inverse variance weighted | 10 | 0.206 | 0.153 | 0.180 |
|  | Simple mode | 10 | 0.008 | 0.293 | 0.979 |
|  | Weighted mode | 10 | 0.002 | 0.309 | 0.995 |
| Melainabacteria |  |  |  |  |  |
|  | MR Egger | 13 | -0.425 | 0.492 | 0.407 |
|  | Weighted median | 13 | 0.083 | 0.212 | 0.695 |
|  | Inverse variance weighted | 13 | -0.041 | 0.152 | 0.787 |
|  | Simple mode | 13 | 0.368 | 0.406 | 0.382 |
|  | Weighted mode | 13 | 0.311 | 0.368 | 0.415 |
| Methanobacteria |  |  |  |  |  |
|  | MR Egger | 12 | -0.990 | 0.457 | 0.056 |
|  | Weighted median | 12 | -0.171 | 0.171 | 0.316 |
|  | Inverse variance weighted | 12 | -0.029 | 0.126 | 0.818 |
|  | Simple mode | 12 | -0.251 | 0.266 | 0.365 |
|  | Weighted mode | 12 | -0.256 | 0.245 | 0.317 |
| Mollicutes |  |  |  |  |  |
|  | MR Egger | 12 | 1.080 | 0.774 | 0.193 |
|  | Weighted median | 12 | -0.125 | 0.294 | 0.670 |
|  | Inverse variance weighted | 12 | -0.077 | 0.244 | 0.751 |
|  | Simple mode | 12 | -0.834 | 0.512 | 0.132 |
|  | Weighted mode | 12 | -0.718 | 0.584 | 0.245 |
| Negativicutes |  |  |  |  |  |
|  | MR Egger | 34 | -1.648 | 1.030 | 0.119 |
|  | Weighted median | 34 | 0.237 | 0.245 | 0.333 |
|  | Inverse variance weighted | 34 | 0.167 | 0.218 | 0.442 |
|  | Simple mode | 34 | -1.108 | 0.803 | 0.177 |
|  | Weighted mode | 34 | -1.051 | 0.701 | 0.143 |
| Verrucomicrobiae |  |  |  |  |  |
|  | MR Egger | 12 | -0.113 | 0.702 | 0.875 |
|  | Weighted median | 12 | 0.104 | 0.279 | 0.708 |
|  | Inverse variance weighted | 12 | 0.258 | 0.199 | 0.194 |
|  | Simple mode | 12 | -0.026 | 0.442 | 0.953 |
|  | Weighted mode | 12 | -0.054 | 0.393 | 0.892 |
| Family |  |  |  |  |  |
| Acidaminococcaceae |  |  |  |  |  |
|  | MR Egger | 8 | -0.699 | 0.650 | 0.323 |
|  | Weighted median | 8 | -0.499 | 0.300 | 0.092 |
|  | Inverse variance weighted | 8 | -0.458 | 0.228 | 0.044 |
|  | Simple mode | 8 | -0.711 | 0.428 | 0.140 |
|  | Weighted mode | 8 | -0.553 | 0.402 | 0.212 |
| Actinomycetaceae |  |  |  |  |  |
|  | MR Egger | 4 | 0.783 | 0.572 | 0.304 |
|  | Weighted median | 4 | -0.116 | 0.324 | 0.721 |
|  | Inverse variance weighted | 4 | 0.000 | 0.262 | 1.000 |
|  | Simple mode | 4 | -0.408 | 0.555 | 0.516 |
|  | Weighted mode | 4 | 0.393 | 0.356 | 0.351 |
| Alcaligenaceae |  |  |  |  |  |
|  | MR Egger | 16 | -1.026 | 0.900 | 0.273 |
|  | Weighted median | 16 | 0.149 | 0.286 | 0.602 |
|  | Inverse variance weighted | 16 | 0.231 | 0.220 | 0.293 |
|  | Simple mode | 16 | 0.101 | 0.464 | 0.831 |
|  | Weighted mode | 16 | 0.111 | 0.454 | 0.810 |
| Bacteroidaceae |  |  |  |  |  |
|  | MR Egger | 12 | 0.499 | 1.278 | 0.705 |
|  | Weighted median | 12 | -0.050 | 0.336 | 0.882 |
|  | Inverse variance weighted | 12 | -0.002 | 0.242 | 0.993 |
|  | Simple mode | 12 | -0.023 | 0.512 | 0.966 |
|  | Weighted mode | 12 | -0.054 | 0.459 | 0.908 |
| BacteroidalesS24.7group |  |  |  |  |  |
|  | MR Egger | 10 | 0.199 | 0.756 | 0.799 |
|  | Weighted median | 10 | -0.460 | 0.230 | 0.046 |
|  | Inverse variance weighted | 10 | -0.379 | 0.178 | 0.033 |
|  | Simple mode | 10 | -0.490 | 0.344 | 0.188 |
|  | Weighted mode | 10 | -0.487 | 0.316 | 0.158 |
| Bifidobacteriaceae |  |  |  |  |  |
|  | MR Egger | 17 | -0.405 | 0.644 | 0.539 |
|  | Weighted median | 17 | -0.032 | 0.252 | 0.896 |
|  | Inverse variance weighted | 17 | 0.082 | 0.175 | 0.639 |
|  | Simple mode | 17 | -0.268 | 0.490 | 0.591 |
|  | Weighted mode | 17 | -0.310 | 0.352 | 0.392 |
| Christensenellaceae |  |  |  |  |  |
|  | MR Egger | 12 | 0.331 | 0.373 | 0.395 |
|  | Weighted median | 12 | 0.366 | 0.262 | 0.163 |
|  | Inverse variance weighted | 12 | 0.293 | 0.191 | 0.126 |
|  | Simple mode | 12 | 0.530 | 0.396 | 0.208 |
|  | Weighted mode | 12 | 0.388 | 0.308 | 0.234 |
| Clostridiaceae1 |  |  |  |  |  |
|  | MR Egger | 11 | -0.782 | 0.803 | 0.355 |
|  | Weighted median | 11 | -0.077 | 0.323 | 0.812 |
|  | Inverse variance weighted | 11 | 0.024 | 0.269 | 0.928 |
|  | Simple mode | 11 | -0.061 | 0.585 | 0.919 |
|  | Weighted mode | 11 | -0.284 | 0.534 | 0.606 |
| ClostridialesvadinBB60group |  |  |  |  |  |
|  | MR Egger | 17 | 0.312 | 0.439 | 0.488 |
|  | Weighted median | 17 | 0.030 | 0.217 | 0.891 |
|  | Inverse variance weighted | 17 | 0.012 | 0.159 | 0.936 |
|  | Simple mode | 17 | 0.207 | 0.396 | 0.609 |
|  | Weighted mode | 17 | 0.086 | 0.360 | 0.814 |
| Coriobacteriaceae |  |  |  |  |  |
|  | MR Egger | 17 | 0.410 | 0.485 | 0.411 |
|  | Weighted median | 17 | 0.346 | 0.276 | 0.210 |
|  | Inverse variance weighted | 17 | 0.300 | 0.193 | 0.121 |
|  | Simple mode | 17 | 0.302 | 0.428 | 0.491 |
|  | Weighted mode | 17 | 0.375 | 0.360 | 0.313 |
| Defluviitaleaceae |  |  |  |  |  |
|  | MR Egger | 12 | -0.495 | 0.623 | 0.445 |
|  | Weighted median | 12 | -0.086 | 0.240 | 0.718 |
|  | Inverse variance weighted | 12 | -0.090 | 0.175 | 0.608 |
|  | Simple mode | 12 | 0.015 | 0.400 | 0.971 |
|  | Weighted mode | 12 | 0.059 | 0.357 | 0.871 |
| Desulfovibrionaceae |  |  |  |  |  |
|  | MR Egger | 12 | 0.154 | 0.551 | 0.785 |
|  | Weighted median | 12 | -0.169 | 0.290 | 0.561 |
|  | Inverse variance weighted | 12 | -0.271 | 0.213 | 0.203 |
|  | Simple mode | 12 | -0.227 | 0.461 | 0.632 |
|  | Weighted mode | 12 | -0.158 | 0.351 | 0.662 |
| Enterobacteriaceae |  |  |  |  |  |
|  | MR Egger | 11 | 0.637 | 1.098 | 0.576 |
|  | Weighted median | 11 | -0.021 | 0.317 | 0.946 |
|  | Inverse variance weighted | 11 | 0.290 | 0.240 | 0.226 |
|  | Simple mode | 11 | -0.181 | 0.544 | 0.746 |
|  | Weighted mode | 11 | -0.190 | 0.508 | 0.716 |
| Erysipelotrichaceae |  |  |  |  |  |
|  | MR Egger | 13 | -0.696 | 1.317 | 0.608 |
|  | Weighted median | 13 | -0.215 | 0.371 | 0.562 |
|  | Inverse variance weighted | 13 | -0.111 | 0.294 | 0.706 |
|  | Simple mode | 13 | -0.532 | 0.679 | 0.448 |
|  | Weighted mode | 13 | -0.560 | 0.640 | 0.399 |
| FamilyXI |  |  |  |  |  |
|  | MR Egger | 10 | -1.012 | 0.572 | 0.115 |
|  | Weighted median | 10 | -0.063 | 0.159 | 0.692 |
|  | Inverse variance weighted | 10 | -0.047 | 0.126 | 0.710 |
|  | Simple mode | 10 | 0.266 | 0.299 | 0.397 |
|  | Weighted mode | 10 | 0.256 | 0.293 | 0.405 |
| FamilyXIII |  |  |  |  |  |
|  | MR Egger | 13 | -0.795 | 1.058 | 0.468 |
|  | Weighted median | 13 | -0.835 | 0.314 | 0.008 |
|  | Inverse variance weighted | 13 | -0.516 | 0.297 | 0.083 |
|  | Simple mode | 13 | -0.801 | 0.521 | 0.150 |
|  | Weighted mode | 13 | -0.835 | 0.404 | 0.061 |
| Lachnospiraceae |  |  |  |  |  |
|  | MR Egger | 17 | -0.950 | 0.826 | 0.268 |
|  | Weighted median | 17 | -0.015 | 0.312 | 0.961 |
|  | Inverse variance weighted | 17 | 0.080 | 0.236 | 0.735 |
|  | Simple mode | 17 | 0.108 | 0.616 | 0.863 |
|  | Weighted mode | 17 | 0.072 | 0.588 | 0.905 |
| Lactobacillaceae |  |  |  |  |  |
|  | MR Egger | 11 | 0.645 | 0.401 | 0.142 |
|  | Weighted median | 11 | 0.201 | 0.212 | 0.344 |
|  | Inverse variance weighted | 11 | 0.205 | 0.150 | 0.171 |
|  | Simple mode | 11 | 0.566 | 0.357 | 0.144 |
|  | Weighted mode | 11 | 0.263 | 0.273 | 0.359 |
| Methanobacteriaceae |  |  |  |  |  |
|  | MR Egger | 12 | -0.990 | 0.457 | 0.056 |
|  | Weighted median | 12 | -0.171 | 0.173 | 0.322 |
|  | Inverse variance weighted | 12 | -0.029 | 0.126 | 0.818 |
|  | Simple mode | 12 | -0.251 | 0.262 | 0.358 |
|  | Weighted mode | 12 | -0.257 | 0.242 | 0.311 |
| Oxalobacteraceae |  |  |  |  |  |
|  | MR Egger | 15 | -0.094 | 0.477 | 0.846 |
|  | Weighted median | 15 | 0.044 | 0.157 | 0.781 |
|  | Inverse variance weighted | 15 | 0.012 | 0.118 | 0.920 |
|  | Simple mode | 15 | 0.016 | 0.249 | 0.951 |
|  | Weighted mode | 15 | 0.016 | 0.236 | 0.948 |
| Pasteurellaceae |  |  |  |  |  |
|  | MR Egger | 19 | -0.129 | 0.306 | 0.677 |
|  | Weighted median | 19 | 0.075 | 0.188 | 0.687 |
|  | Inverse variance weighted | 19 | 0.145 | 0.132 | 0.270 |
|  | Simple mode | 19 | 0.165 | 0.300 | 0.589 |
|  | Weighted mode | 19 | 0.051 | 0.269 | 0.852 |
| Peptococcaceae |  |  |  |  |  |
|  | MR Egger | 9 | -0.154 | 0.805 | 0.854 |
|  | Weighted median | 9 | 0.107 | 0.310 | 0.730 |
|  | Inverse variance weighted | 9 | 0.023 | 0.274 | 0.932 |
|  | Simple mode | 9 | 0.581 | 0.594 | 0.357 |
|  | Weighted mode | 9 | 0.401 | 0.516 | 0.460 |
| Peptostreptococcaceae |  |  |  |  |  |
|  | MR Egger | 15 | -0.410 | 0.415 | 0.342 |
|  | Weighted median | 15 | -0.114 | 0.245 | 0.643 |
|  | Inverse variance weighted | 15 | 0.075 | 0.179 | 0.673 |
|  | Simple mode | 15 | 0.121 | 0.418 | 0.777 |
|  | Weighted mode | 15 | -0.133 | 0.357 | 0.715 |
| Porphyromonadaceae |  |  |  |  |  |
|  | MR Egger | 10 | 0.164 | 1.275 | 0.901 |
|  | Weighted median | 10 | -0.300 | 0.371 | 0.418 |
|  | Inverse variance weighted | 10 | -0.148 | 0.286 | 0.605 |
|  | Simple mode | 10 | -0.542 | 0.549 | 0.349 |
|  | Weighted mode | 10 | -0.488 | 0.557 | 0.403 |
| Prevotellaceae |  |  |  |  |  |
|  | MR Egger | 18 | 1.080 | 0.575 | 0.079 |
|  | Weighted median | 18 | 0.218 | 0.251 | 0.386 |
|  | Inverse variance weighted | 18 | 0.098 | 0.181 | 0.586 |
|  | Simple mode | 18 | 0.221 | 0.457 | 0.635 |
|  | Weighted mode | 18 | 0.471 | 0.429 | 0.287 |
| Rhodospirillaceae |  |  |  |  |  |
|  | MR Egger | 17 | -1.126 | 0.582 | 0.072 |
|  | Weighted median | 17 | -0.011 | 0.203 | 0.957 |
|  | Inverse variance weighted | 17 | -0.078 | 0.141 | 0.578 |
|  | Simple mode | 17 | 0.004 | 0.372 | 0.993 |
|  | Weighted mode | 17 | 0.026 | 0.361 | 0.944 |
| Rikenellaceae |  |  |  |  |  |
|  | MR Egger | 18 | 0.342 | 0.611 | 0.583 |
|  | Weighted median | 18 | 0.212 | 0.262 | 0.420 |
|  | Inverse variance weighted | 18 | 0.213 | 0.195 | 0.276 |
|  | Simple mode | 18 | -0.052 | 0.475 | 0.914 |
|  | Weighted mode | 18 | -0.043 | 0.465 | 0.926 |
| Ruminococcaceae |  |  |  |  |  |
|  | MR Egger | 12 | 0.577 | 0.491 | 0.267 |
|  | Weighted median | 12 | 0.392 | 0.302 | 0.195 |
|  | Inverse variance weighted | 12 | 0.301 | 0.223 | 0.178 |
|  | Simple mode | 12 | 0.214 | 0.441 | 0.637 |
|  | Weighted mode | 12 | 0.486 | 0.399 | 0.248 |
| Streptococcaceae |  |  |  |  |  |
|  | MR Egger | 17 | -0.843 | 0.728 | 0.265 |
|  | Weighted median | 17 | -0.181 | 0.278 | 0.515 |
|  | Inverse variance weighted | 17 | -0.317 | 0.196 | 0.107 |
|  | Simple mode | 17 | 0.048 | 0.455 | 0.917 |
|  | Weighted mode | 17 | 0.067 | 0.443 | 0.881 |
| Veillonellaceae |  |  |  |  |  |
|  | MR Egger | 21 | 0.008 | 0.333 | 0.982 |
|  | Weighted median | 21 | 0.043 | 0.228 | 0.850 |
|  | Inverse variance weighted | 21 | -0.009 | 0.161 | 0.957 |
|  | Simple mode | 21 | 0.031 | 0.340 | 0.929 |
|  | Weighted mode | 21 | 0.065 | 0.270 | 0.813 |
| Verrucomicrobiaceae |  |  |  |  |  |
|  | MR Egger | 12 | -0.114 | 0.702 | 0.875 |
|  | Weighted median | 12 | 0.104 | 0.279 | 0.708 |
|  | Inverse variance weighted | 12 | 0.258 | 0.199 | 0.194 |
|  | Simple mode | 12 | -0.027 | 0.435 | 0.952 |
|  | Weighted mode | 12 | -0.054 | 0.389 | 0.891 |
| Victivallaceae |  |  |  |  |  |
|  | MR Egger | 14 | -0.612 | 0.522 | 0.264 |
|  | Weighted median | 14 | 0.023 | 0.143 | 0.874 |
|  | Inverse variance weighted | 14 | 0.106 | 0.105 | 0.314 |
|  | Simple mode | 14 | -0.049 | 0.235 | 0.838 |
|  | Weighted mode | 14 | -0.045 | 0.241 | 0.856 |
| Genus |  |  |  |  |  |
| Clostridiuminnocuumgroup |  |  |  |  |  |
|  | MR Egger | 10 | 1.221 | 0.673 | 0.107 |
|  | Weighted median | 10 | 0.094 | 0.174 | 0.590 |
|  | Inverse variance weighted | 10 | 0.214 | 0.130 | 0.100 |
|  | Simple mode | 10 | -0.028 | 0.294 | 0.925 |
|  | Weighted mode | 10 | -0.038 | 0.273 | 0.893 |
| Eubacteriumbrachygroup |  |  |  |  |  |
|  | MR Egger | 11 | 0.306 | 0.476 | 0.536 |
|  | Weighted median | 11 | 0.016 | 0.184 | 0.932 |
|  | Inverse variance weighted | 11 | -0.024 | 0.128 | 0.850 |
|  | Simple mode | 11 | 0.048 | 0.298 | 0.877 |
|  | Weighted mode | 11 | 0.069 | 0.308 | 0.827 |
| Eubacteriumcoprostanoligenesgroup |  |  |  |  |  |
|  | MR Egger | 14 | -1.128 | 1.244 | 0.382 |
|  | Weighted median | 14 | -0.111 | 0.350 | 0.752 |
|  | Inverse variance weighted | 14 | 0.111 | 0.318 | 0.610 |
|  | Simple mode | 14 | -0.312 | 0.600 | 0.611 |
|  | Weighted mode | 14 | -0.283 | 0.482 | 0.567 |
| Eubacteriumeligensgroup |  |  |  |  |  |
|  | MR Egger | 10 | -0.995 | 0.763 | 0.228 |
|  | Weighted median | 10 | -0.321 | 0.332 | 0.334 |
|  | Inverse variance weighted | 10 | -0.281 | 0.279 | 0.313 |
|  | Simple mode | 10 | 0.302 | 0.597 | 0.625 |
|  | Weighted mode | 10 | -0.359 | 0.494 | 0.485 |
| Eubacteriumfissicatenagroup |  |  |  |  |  |
|  | MR Egger | 9 | -0.573 | 0.734 | 0.461 |
|  | Weighted median | 9 | -0.277 | 0.178 | 0.121 |
|  | Inverse variance weighted | 9 | -0.216 | 0.141 | 0.127 |
|  | Simple mode | 9 | -0.319 | 0.263 | 0.260 |
|  | Weighted mode | 9 | -0.322 | 0.271 | 0.269 |
| Eubacteriumhalliigroup |  |  |  |  |  |
|  | MR Egger | 14 | 0.643 | 0.407 | 0.140 |
|  | Weighted median | 14 | 0.076 | 0.273 | 0.782 |
|  | Inverse variance weighted | 14 | 0.031 | 0.196 | 0.876 |
|  | Simple mode | 14 | 0.257 | 0.473 | 0.596 |
|  | Weighted mode | 14 | 0.297 | 0.412 | 0.483 |
| Eubacteriumnodatumgroup |  |  |  |  |  |
|  | MR Egger | 11 | -0.542 | 0.514 | 0.319 |
|  | Weighted median | 11 | 0.001 | 0.155 | 0.996 |
|  | Inverse variance weighted | 11 | 0.044 | 0.115 | 0.702 |
|  | Simple mode | 11 | -0.069 | 0.249 | 0.789 |
|  | Weighted mode | 11 | -0.038 | 0.236 | 0.874 |
| Eubacteriumoxidoreducensgroup |  |  |  |  |  |
|  | MR Egger | 6 | 0.567 | 0.815 | 0.525 |
|  | Weighted median | 6 | -0.071 | 0.252 | 0.778 |
|  | Inverse variance weighted | 6 | -0.096 | 0.200 | 0.632 |
|  | Simple mode | 6 | 0.080 | 0.415 | 0.854 |
|  | Weighted mode | 6 | 0.051 | 0.396 | 0.903 |
| Eubacteriumrectalegroup |  |  |  |  |  |
|  | MR Egger | 11 | 0.255 | 0.635 | 0.697 |
|  | Weighted median | 11 | -0.054 | 0.305 | 0.861 |
|  | Inverse variance weighted | 11 | -0.107 | 0.231 | 0.641 |
|  | Simple mode | 11 | -0.110 | 0.483 | 0.824 |
|  | Weighted mode | 11 | -0.073 | 0.444 | 0.872 |
| Eubacteriumruminantiumgroup |  |  |  |  |  |
|  | MR Egger | 19 | -0.701 | 0.421 | 0.114 |
|  | Weighted median | 19 | -0.492 | 0.186 | 0.008 |
|  | Inverse variance weighted | 19 | -0.383 | 0.127 | 0.002 |
|  | Simple mode | 19 | -0.710 | 0.368 | 0.070 |
|  | Weighted mode | 19 | -0.669 | 0.347 | 0.070 |
| Eubacteriumventriosumgroup |  |  |  |  |  |
|  | MR Egger | 16 | 1.232 | 0.891 | 0.188 |
|  | Weighted median | 16 | -0.455 | 0.261 | 0.080 |
|  | Inverse variance weighted | 16 | -0.309 | 0.198 | 0.118 |
|  | Simple mode | 16 | -0.485 | 0.446 | 0.294 |
|  | Weighted mode | 16 | -0.492 | 0.477 | 0.319 |
| Eubacteriumxylanophilumgroup |  |  |  |  |  |
|  | MR Egger | 12 | -0.393 | 0.613 | 0.536 |
|  | Weighted median | 12 | -0.327 | 0.271 | 0.228 |
|  | Inverse variance weighted | 12 | -0.142 | 0.200 | 0.477 |
|  | Simple mode | 12 | -0.570 | 0.440 | 0.222 |
|  | Weighted mode | 12 | -0.540 | 0.451 | 0.257 |
| Ruminococcusgauvreauiigroup |  |  |  |  |  |
|  | MR Egger | 13 | 0.701 | 0.937 | 0.471 |
|  | Weighted median | 13 | 0.465 | 0.274 | 0.089 |
|  | Inverse variance weighted | 13 | 0.463 | 0.208 | 0.026 |
|  | Simple mode | 13 | 0.486 | 0.407 | 0.256 |
|  | Weighted mode | 13 | 0.502 | 0.404 | 0.237 |
| Ruminococcusgnavusgroup |  |  |  |  |  |
|  | MR Egger | 11 | 1.229 | 0.900 | 0.205 |
|  | Weighted median | 11 | 0.166 | 0.224 | 0.458 |
|  | Inverse variance weighted | 11 | -0.039 | 0.198 | 0.844 |
|  | Simple mode | 11 | 0.263 | 0.406 | 0.531 |
|  | Weighted mode | 11 | 0.295 | 0.384 | 0.461 |
| Ruminococcustorquesgroup |  |  |  |  |  |
|  | MR Egger | 13 | 0.062 | 0.894 | 0.946 |
|  | Weighted median | 13 | 0.209 | 0.337 | 0.536 |
|  | Inverse variance weighted | 13 | -0.138 | 0.249 | 0.580 |
|  | Simple mode | 13 | 0.417 | 0.584 | 0.489 |
|  | Weighted mode | 13 | 0.376 | 0.561 | 0.516 |
| Actinomyces |  |  |  |  |  |
|  | MR Egger | 8 | -0.386 | 0.834 | 0.660 |
|  | Weighted median | 8 | 0.264 | 0.281 | 0.347 |
|  | Inverse variance weighted | 8 | -0.103 | 0.307 | 0.737 |
|  | Simple mode | 8 | 0.331 | 0.522 | 0.546 |
|  | Weighted mode | 8 | 0.395 | 0.351 | 0.297 |
| Adlercreutzia |  |  |  |  |  |
|  | MR Egger | 12 | 1.064 | 0.839 | 0.233 |
|  | Weighted median | 12 | 0.033 | 0.244 | 0.893 |
|  | Inverse variance weighted | 12 | 0.227 | 0.173 | 0.188 |
|  | Simple mode | 12 | -0.099 | 0.401 | 0.809 |
|  | Weighted mode | 12 | -0.144 | 0.370 | 0.705 |
| Akkermansia |  |  |  |  |  |
|  | MR Egger | 12 | -0.110 | 0.702 | 0.879 |
|  | Weighted median | 12 | 0.105 | 0.282 | 0.709 |
|  | Inverse variance weighted | 12 | 0.258 | 0.199 | 0.194 |
|  | Simple mode | 12 | -0.029 | 0.432 | 0.948 |
|  | Weighted mode | 12 | -0.047 | 0.392 | 0.906 |
| Alistipes |  |  |  |  |  |
|  | MR Egger | 14 | 0.722 | 1.592 | 0.658 |
|  | Weighted median | 14 | -0.529 | 0.363 | 0.145 |
|  | Inverse variance weighted | 14 | -0.267 | 0.311 | 0.390 |
|  | Simple mode | 14 | -0.668 | 0.570 | 0.262 |
|  | Weighted mode | 14 | -0.655 | 0.601 | 0.296 |
| Allisonella |  |  |  |  |  |
|  | MR Egger | 9 | 1.280 | 0.780 | 0.145 |
|  | Weighted median | 9 | -0.007 | 0.158 | 0.962 |
|  | Inverse variance weighted | 9 | -0.042 | 0.127 | 0.742 |
|  | Simple mode | 9 | -0.042 | 0.258 | 0.876 |
|  | Weighted mode | 9 | -0.032 | 0.253 | 0.900 |
| Alloprevotella |  |  |  |  |  |
|  | MR Egger | 6 | 2.983 | 1.338 | 0.090 |
|  | Weighted median | 6 | 0.079 | 0.209 | 0.705 |
|  | Inverse variance weighted | 6 | 0.131 | 0.189 | 0.487 |
|  | Simple mode | 6 | -0.122 | 0.375 | 0.758 |
|  | Weighted mode | 6 | -0.160 | 0.368 | 0.682 |
| Anaerofilum |  |  |  |  |  |
|  | MR Egger | 11 | -0.720 | 0.634 | 0.286 |
|  | Weighted median | 11 | -0.357 | 0.192 | 0.063 |
|  | Inverse variance weighted | 11 | -0.377 | 0.139 | 0.007 |
|  | Simple mode | 11 | -0.491 | 0.336 | 0.174 |
|  | Weighted mode | 11 | 0.501 | 0.313 | 0.140 |
| Anaerostipes |  |  |  |  |  |
|  | MR Egger | 15 | -0.461 | 0.732 | 0.540 |
|  | Weighted median | 15 | -0.344 | 0.316 | 0.276 |
|  | Inverse variance weighted | 15 | -0.224 | 0.236 | 0.344 |
|  | Simple mode | 15 | -0.507 | 0.519 | 0.345 |
|  | Weighted mode | 15 | -0.486 | 0.541 | 0.384 |
| Anaerotruncus |  |  |  |  |  |
|  | MR Egger | 15 | 1.046 | 0.656 | 0.135 |
|  | Weighted median | 15 | 0.251 | 0.315 | 0.427 |
|  | Inverse variance weighted | 15 | 0.346 | 0.218 | 0.112 |
|  | Simple mode | 15 | 0.082 | 0.567 | 0.888 |
|  | Weighted mode | 15 | 0.093 | 0.537 | 0.865 |
| Bacteroides |  |  |  |  |  |
|  | MR Egger | 12 | 0.499 | 1.278 | 0.705 |
|  | Weighted median | 12 | -0.050 | 0.316 | 0.874 |
|  | Inverse variance weighted | 12 | -0.002 | 0.242 | 0.993 |
|  | Simple mode | 12 | -0.023 | 0.504 | 0.965 |
|  | Weighted mode | 12 | -0.054 | 0.440 | 0.904 |
| Barnesiella |  |  |  |  |  |
|  | MR Egger | 16 | -0.934 | 0.825 | 0.277 |
|  | Weighted median | 16 | -0.053 | 0.261 | 0.840 |
|  | Inverse variance weighted | 16 | -0.159 | 0.191 | 0.406 |
|  | Simple mode | 16 | 0.091 | 0.454 | 0.845 |
|  | Weighted mode | 16 | -0.012 | 0.463 | 0.980 |
| Bifidobacterium |  |  |  |  |  |
|  | MR Egger | 15 | -0.464 | 0.463 | 0.335 |
|  | Weighted median | 15 | 0.107 | 0.253 | 0.673 |
|  | Inverse variance weighted | 15 | 0.175 | 0.176 | 0.319 |
|  | Simple mode | 15 | 0.660 | 0.428 | 0.145 |
|  | Weighted mode | 15 | -0.220 | 0.340 | 0.530 |
| Bilophila |  |  |  |  |  |
|  | MR Egger | 16 | -0.277 | 0.785 | 0.730 |
|  | Weighted median | 16 | -0.022 | 0.284 | 0.937 |
|  | Inverse variance weighted | 16 | 0.012 | 0.188 | 0.948 |
|  | Simple mode | 16 | 0.111 | 0.530 | 0.837 |
|  | Weighted mode | 16 | 0.409 | 0.518 | 0.441 |
| Blautia |  |  |  |  |  |
|  | MR Egger | 13 | -0.787 | 0.778 | 0.333 |
|  | Weighted median | 13 | 0.446 | 0.373 | 0.232 |
|  | Inverse variance weighted | 13 | 0.022 | 0.300 | 0.941 |
|  | Simple mode | 13 | 0.747 | 0.784 | 0.360 |
|  | Weighted mode | 13 | 0.693 | 0.716 | 0.352 |
| Butyricicoccus |  |  |  |  |  |
|  | MR Egger | 9 | -0.335 | 0.509 | 0.532 |
|  | Weighted median | 9 | -0.052 | 0.332 | 0.875 |
|  | Inverse variance weighted | 9 | -0.007 | 0.254 | 0.979 |
|  | Simple mode | 9 | 0.141 | 0.500 | 0.785 |
|  | Weighted mode | 9 | -0.079 | 0.405 | 0.850 |
| Butyricimonas |  |  |  |  |  |
|  | MR Egger | 17 | 0.110 | 0.795 | 0.892 |
|  | Weighted median | 17 | -0.174 | 0.255 | 0.497 |
|  | Inverse variance weighted | 17 | -0.067 | 0.195 | 0.733 |
|  | Simple mode | 17 | 0.465 | 0.526 | 0.390 |
|  | Weighted mode | 17 | -0.355 | 0.425 | 0.416 |
| Butyrivibrio |  |  |  |  |  |
|  | MR Egger | 16 | -0.016 | 0.402 | 0.970 |
|  | Weighted median | 16 | -0.152 | 0.118 | 0.196 |
|  | Inverse variance weighted | 16 | -0.186 | 0.094 | 0.047 |
|  | Simple mode | 16 | -0.095 | 0.221 | 0.674 |
|  | Weighted mode | 16 | -0.115 | 0.218 | 0.606 |
| CandidatusSoleaferrea |  |  |  |  |  |
|  | MR Egger | 15 | -0.140 | 0.601 | 0.820 |
|  | Weighted median | 15 | -0.233 | 0.180 | 0.196 |
|  | Inverse variance weighted | 15 | -0.108 | 0.133 | 0.434 |
|  | Simple mode | 15 | -0.265 | 0.304 | 0.398 |
|  | Weighted mode | 15 | -0.265 | 0.302 | 0.395 |
| Catenibacterium |  |  |  |  |  |
|  | MR Egger | 4 | 0.449 | 3.719 | 0.915 |
|  | Weighted median | 4 | 0.179 | 0.253 | 0.478 |
|  | Inverse variance weighted | 4 | 0.009 | 0.243 | 0.970 |
|  | Simple mode | 4 | 0.210 | 0.337 | 0.578 |
|  | Weighted mode | 4 | 0.206 | 0.334 | 0.581 |
| ChristensenellaceaeR.7group |  |  |  |  |  |
|  | MR Egger | 10 | 1.564 | 0.965 | 0.144 |
|  | Weighted median | 10 | 0.024 | 0.364 | 0.946 |
|  | Inverse variance weighted | 10 | 0.199 | 0.272 | 0.465 |
|  | Simple mode | 10 | -0.243 | 0.617 | 0.703 |
|  | Weighted mode | 10 | -0.243 | 0.567 | 0.679 |
| Clostridiumsensustricto1 |  |  |  |  |  |
|  | MR Egger | 9 | -0.903 | 0.598 | 0.175 |
|  | Weighted median | 9 | -0.152 | 0.327 | 0.641 |
|  | Inverse variance weighted | 9 | -0.258 | 0.232 | 0.265 |
|  | Simple mode | 9 | 0.130 | 0.572 | 0.826 |
|  | Weighted mode | 9 | 0.164 | 0.536 | 0.768 |
| Collinsella |  |  |  |  |  |
|  | MR Egger | 12 | -0.848 | 0.998 | 0.415 |
|  | Weighted median | 12 | 0.242 | 0.322 | 0.453 |
|  | Inverse variance weighted | 12 | 0.053 | 0.242 | 0.828 |
|  | Simple mode | 12 | 0.179 | 0.611 | 0.775 |
|  | Weighted mode | 12 | 0.230 | 0.554 | 0.686 |
| Coprobacter |  |  |  |  |  |
|  | MR Egger | 14 | 0.055 | 0.455 | 0.906 |
|  | Weighted median | 14 | -0.015 | 0.188 | 0.934 |
|  | Inverse variance weighted | 14 | 0.095 | 0.140 | 0.496 |
|  | Simple mode | 14 | -0.037 | 0.308 | 0.907 |
|  | Weighted mode | 14 | -0.023 | 0.280 | 0.936 |
| Coprococcus1 |  |  |  |  |  |
|  | MR Egger | 14 | -0.145 | 0.538 | 0.792 |
|  | Weighted median | 14 | -0.157 | 0.285 | 0.581 |
|  | Inverse variance weighted | 14 | 0.007 | 0.208 | 0.974 |
|  | Simple mode | 14 | -0.256 | 0.463 | 0.590 |
|  | Weighted mode | 14 | -0.231 | 0.408 | 0.581 |
| Coprococcus2 |  |  |  |  |  |
|  | MR Egger | 10 | 0.531 | 1.197 | 0.670 |
|  | Weighted median | 10 | 0.211 | 0.276 | 0.445 |
|  | Inverse variance weighted | 10 | 0.028 | 0.218 | 0.898 |
|  | Simple mode | 10 | 0.283 | 0.494 | 0.581 |
|  | Weighted mode | 10 | 0.302 | 0.524 | 0.578 |
| Coprococcus3 |  |  |  |  |  |
|  | MR Egger | 10 | -1.417 | 1.628 | 0.410 |
|  | Weighted median | 10 | -0.099 | 0.362 | 0.785 |
|  | Inverse variance weighted | 10 | 0.002 | 0.272 | 0.994 |
|  | Simple mode | 10 | -0.477 | 0.665 | 0.491 |
|  | Weighted mode | 10 | -0.477 | 0.606 | 0.451 |
| DefluviitaleaceaeUCG011 |  |  |  |  |  |
|  | MR Egger | 10 | -0.504 | 0.735 | 0.512 |
|  | Weighted median | 10 | 0.067 | 0.274 | 0.807 |
|  | Inverse variance weighted | 10 | -0.020 | 0.194 | 0.918 |
|  | Simple mode | 10 | 0.233 | 0.409 | 0.583 |
|  | Weighted mode | 10 | 0.248 | 0.375 | 0.525 |
| Desulfovibrio |  |  |  |  |  |
|  | MR Egger | 11 | -0.286 | 0.897 | 0.758 |
|  | Weighted median | 11 | 0.197 | 0.299 | 0.510 |
|  | Inverse variance weighted | 11 | 0.214 | 0.289 | 0.458 |
|  | Simple mode | 11 | -0.503 | 0.586 | 0.411 |
|  | Weighted mode | 11 | -0.466 | 0.556 | 0.422 |
| Dialister |  |  |  |  |  |
|  | MR Egger | 12 | -1.541 | 0.883 | 0.112 |
|  | Weighted median | 12 | 0.077 | 0.293 | 0.791 |
|  | Inverse variance weighted | 12 | 0.127 | 0.212 | 0.547 |
|  | Simple mode | 12 | 0.096 | 0.512 | 0.855 |
|  | Weighted mode | 12 | 0.058 | 0.476 | 0.905 |
| Dorea |  |  |  |  |  |
|  | MR Egger | 12 | 0.275 | 0.736 | 0.717 |
|  | Weighted median | 12 | -0.208 | 0.353 | 0.557 |
|  | Inverse variance weighted | 12 | -0.427 | 0.250 | 0.088 |
|  | Simple mode | 12 | -0.103 | 0.575 | 0.861 |
|  | Weighted mode | 12 | -0.129 | 0.501 | 0.802 |
| Eggerthella |  |  |  |  |  |
|  | MR Egger | 10 | -2.056 | 0.705 | 0.019 |
|  | Weighted median | 10 | 0.487 | 0.223 | 0.029 |
|  | Inverse variance weighted | 10 | 0.329 | 0.218 | 0.131 |
|  | Simple mode | 10 | 0.514 | 0.350 | 0.176 |
|  | Weighted mode | 10 | 0.530 | 0.354 | 0.168 |
| Eisenbergiella |  |  |  |  |  |
|  | MR Egger | 12 | 2.284 | 1.020 | 0.049 |
|  | Weighted median | 12 | 0.255 | 0.185 | 0.168 |
|  | Inverse variance weighted | 12 | 0.287 | 0.138 | 0.037 |
|  | Simple mode | 12 | 0.100 | 0.297 | 0.742 |
|  | Weighted mode | 12 | 0.155 | 0.301 | 0.617 |
| Enterorhabdus |  |  |  |  |  |
|  | MR Egger | 9 | -0.345 | 0.535 | 0.539 |
|  | Weighted median | 9 | 0.277 | 0.238 | 0.244 |
|  | Inverse variance weighted | 9 | 0.122 | 0.183 | 0.505 |
|  | Simple mode | 9 | 0.364 | 0.374 | 0.359 |
|  | Weighted mode | 9 | 0.313 | 0.380 | 0.433 |
| Erysipelatoclostridium |  |  |  |  |  |
|  | MR Egger | 16 | 0.297 | 0.635 | 0.647 |
|  | Weighted median | 16 | 0.141 | 0.212 | 0.507 |
|  | Inverse variance weighted | 16 | 0.058 | 0.157 | 0.712 |
|  | Simple mode | 16 | 0.174 | 0.392 | 0.664 |
|  | Weighted mode | 16 | 0.180 | 0.384 | 0.646 |
| ErysipelotrichaceaeUCG003 |  |  |  |  |  |
|  | MR Egger | 17 | -0.640 | 0.507 | 0.226 |
|  | Weighted median | 17 | -0.689 | 0.258 | 0.008 |
|  | Inverse variance weighted | 17 | -0.519 | 0.183 | 0.005 |
|  | Simple mode | 17 | -0.796 | 0.427 | 0.081 |
|  | Weighted mode | 17 | -0.796 | 0.434 | 0.085 |
| Escherichia.Shigella |  |  |  |  |  |
|  | MR Egger | 15 | 0.154 | 0.572 | 0.793 |
|  | Weighted median | 15 | -0.219 | 0.250 | 0.380 |
|  | Inverse variance weighted | 15 | -0.122 | 0.186 | 0.511 |
|  | Simple mode | 15 | -0.569 | 0.488 | 0.262 |
|  | Weighted mode | 15 | -0.554 | 0.481 | 0.269 |
| Faecalibacterium |  |  |  |  |  |
|  | MR Egger | 13 | 0.746 | 0.389 | 0.082 |
|  | Weighted median | 13 | 0.121 | 0.279 | 0.665 |
|  | Inverse variance weighted | 13 | 0.182 | 0.199 | 0.362 |
|  | Simple mode | 13 | -0.237 | 0.488 | 0.637 |
|  | Weighted mode | 13 | 0.099 | 0.460 | 0.833 |
| FamilyXIIIAD3011group |  |  |  |  |  |
|  | MR Egger | 15 | 0.759 | 0.980 | 0.452 |
|  | Weighted median | 15 | 0.095 | 0.267 | 0.723 |
|  | Inverse variance weighted | 15 | -0.118 | 0.201 | 0.557 |
|  | Simple mode | 15 | 0.316 | 0.478 | 0.519 |
|  | Weighted mode | 15 | 0.303 | 0.448 | 0.510 |
| FamilyXIIIUCG001 |  |  |  |  |  |
|  | MR Egger | 10 | 1.558 | 0.729 | 0.065 |
|  | Weighted median | 10 | 0.264 | 0.312 | 0.399 |
|  | Inverse variance weighted | 10 | 0.368 | 0.226 | 0.104 |
|  | Simple mode | 10 | 0.199 | 0.491 | 0.694 |
|  | Weighted mode | 10 | 0.160 | 0.511 | 0.762 |
| Flavonifractor |  |  |  |  |  |
|  | MR Egger | 8 | 0.866 | 0.817 | 0.330 |
|  | Weighted median | 8 | -0.102 | 0.299 | 0.734 |
|  | Inverse variance weighted | 8 | -0.124 | 0.226 | 0.584 |
|  | Simple mode | 8 | -0.112 | 0.473 | 0.820 |
|  | Weighted mode | 8 | 0.053 | 0.472 | 0.914 |
| Fusicatenibacter |  |  |  |  |  |
|  | MR Egger | 19 | 0.078 | 0.737 | 0.917 |
|  | Weighted median | 19 | 0.012 | 0.285 | 0.965 |
|  | Inverse variance weighted | 19 | -0.101 | 0.198 | 0.611 |
|  | Simple mode | 19 | 0.067 | 0.525 | 0.900 |
|  | Weighted mode | 19 | 0.046 | 0.468 | 0.922 |
| Gordonibacter |  |  |  |  |  |
|  | MR Egger | 14 | -0.491 | 0.411 | 0.255 |
|  | Weighted median | 14 | 0.094 | 0.133 | 0.477 |
|  | Inverse variance weighted | 14 | 0.013 | 0.104 | 0.897 |
|  | Simple mode | 14 | 0.151 | 0.223 | 0.511 |
|  | Weighted mode | 14 | 0.158 | 0.250 | 0.538 |
| Haemophilus |  |  |  |  |  |
|  | MR Egger | 14 | 0.154 | 0.368 | 0.682 |
|  | Weighted median | 14 | -0.017 | 0.194 | 0.930 |
|  | Inverse variance weighted | 14 | 0.114 | 0.150 | 0.450 |
|  | Simple mode | 14 | -0.079 | 0.334 | 0.817 |
|  | Weighted mode | 14 | -0.065 | 0.299 | 0.830 |
| Holdemanella |  |  |  |  |  |
|  | MR Egger | 11 | 0.050 | 0.441 | 0.912 |
|  | Weighted median | 11 | 0.189 | 0.201 | 0.347 |
|  | Inverse variance weighted | 11 | 0.198 | 0.154 | 0.197 |
|  | Simple mode | 11 | 0.429 | 0.340 | 0.235 |
|  | Weighted mode | 11 | 0.433 | 0.345 | 0.238 |
| Holdemania |  |  |  |  |  |
|  | MR Egger | 18 | -0.068 | 0.472 | 0.887 |
|  | Weighted median | 18 | -0.010 | 0.199 | 0.960 |
|  | Inverse variance weighted | 18 | -0.027 | 0.152 | 0.861 |
|  | Simple mode | 18 | -0.053 | 0.353 | 0.882 |
|  | Weighted mode | 18 | -0.062 | 0.306 | 0.841 |
| Howardella |  |  |  |  |  |
|  | MR Egger | 11 | -0.428 | 0.542 | 0.450 |
|  | Weighted median | 11 | -0.270 | 0.159 | 0.090 |
|  | Inverse variance weighted | 11 | -0.188 | 0.124 | 0.129 |
|  | Simple mode | 11 | -0.507 | 0.294 | 0.115 |
|  | Weighted mode | 11 | -0.452 | 0.267 | 0.122 |
| Hungatella |  |  |  |  |  |
|  | MR Egger | 5 | -0.369 | 1.164 | 0.772 |
|  | Weighted median | 5 | 0.025 | 0.240 | 0.917 |
|  | Inverse variance weighted | 5 | 0.120 | 0.190 | 0.531 |
|  | Simple mode | 5 | -0.033 | 0.322 | 0.923 |
|  | Weighted mode | 5 | -0.062 | 0.291 | 0.843 |
| Intestinibacter |  |  |  |  |  |
|  | MR Egger | 15 | -0.398 | 0.588 | 0.511 |
|  | Weighted median | 15 | 0.044 | 0.256 | 0.862 |
|  | Inverse variance weighted | 15 | -0.046 | 0.181 | 0.801 |
|  | Simple mode | 15 | 0.028 | 0.435 | 0.950 |
|  | Weighted mode | 15 | 0.010 | 0.428 | 0.981 |
| Intestinimonas |  |  |  |  |  |
|  | MR Egger | 19 | 0.681 | 0.449 | 0.147 |
|  | Weighted median | 19 | 0.225 | 0.220 | 0.306 |
|  | Inverse variance weighted | 19 | 0.084 | 0.161 | 0.601 |
|  | Simple mode | 19 | 0.188 | 0.395 | 0.640 |
|  | Weighted mode | 19 | 0.277 | 0.342 | 0.429 |
| Lachnoclostridium |  |  |  |  |  |
|  | MR Egger | 15 | -0.160 | 0.908 | 0.863 |
|  | Weighted median | 15 | -0.024 | 0.324 | 0.940 |
|  | Inverse variance weighted | 15 | 0.170 | 0.257 | 0.508 |
|  | Simple mode | 15 | -0.166 | 0.575 | 0.778 |
|  | Weighted mode | 15 | -0.098 | 0.500 | 0.847 |
| Lachnospira |  |  |  |  |  |
|  | MR Egger | 7 | -1.010 | 1.764 | 0.592 |
|  | Weighted median | 7 | -0.035 | 0.400 | 0.931 |
|  | Inverse variance weighted | 7 | 0.064 | 0.324 | 0.844 |
|  | Simple mode | 7 | -0.193 | 0.607 | 0.761 |
|  | Weighted mode | 7 | -0.274 | 0.578 | 0.653 |
| LachnospiraceaeFCS020group |  |  |  |  |  |
|  | MR Egger | 16 | -0.181 | 0.480 | 0.712 |
|  | Weighted median | 16 | -0.102 | 0.254 | 0.688 |
|  | Inverse variance weighted | 16 | 0.109 | 0.179 | 0.543 |
|  | Simple mode | 16 | -0.213 | 0.419 | 0.619 |
|  | Weighted mode | 16 | -0.178 | 0.384 | 0.650 |
| LachnospiraceaeNC2004group |  |  |  |  |  |
|  | MR Egger | 10 | -0.157 | 0.852 | 0.858 |
|  | Weighted median | 10 | 0.050 | 0.243 | 0.836 |
|  | Inverse variance weighted | 10 | -0.156 | 0.193 | 0.420 |
|  | Simple mode | 10 | 0.138 | 0.381 | 0.727 |
|  | Weighted mode | 10 | 0.131 | 0.340 | 0.710 |
| LachnospiraceaeND3007group |  |  |  |  |  |
|  | MR Egger | 3 | -13.811 | 11.501 | 0.442 |
|  | Weighted median | 3 | 0.643 | 0.706 | 0.927 |
|  | Inverse variance weighted | 3 | 0.569 | 0.756 | 0.940 |
|  | Simple mode | 3 | -0.041 | 0.938 | 0.969 |
|  | Weighted mode | 3 | 0.091 | 0.923 | 0.930 |
| LachnospiraceaeNK4A136group |  |  |  |  |  |
|  | MR Egger | 16 | -0.098 | 0.368 | 0.795 |
|  | Weighted median | 16 | 0.145 | 0.258 | 0.575 |
|  | Inverse variance weighted | 16 | 0.286 | 0.179 | 0.110 |
|  | Simple mode | 16 | 0.234 | 0.414 | 0.580 |
|  | Weighted mode | 16 | 0.150 | 0.287 | 0.609 |
| LachnospiraceaeUCG001 |  |  |  |  |  |
|  | MR Egger | 16 | 1.034 | 0.703 | 0.163 |
|  | Weighted median | 16 | 0.069 | 0.217 | 0.751 |
|  | Inverse variance weighted | 16 | 0.172 | 0.157 | 0.274 |
|  | Simple mode | 16 | 0.051 | 0.399 | 0.899 |
|  | Weighted mode | 16 | 0.044 | 0.369 | 0.906 |
| LachnospiraceaeUCG004 |  |  |  |  |  |
|  | MR Egger | 13 | 0.475 | 1.086 | 0.670 |
|  | Weighted median | 13 | -0.246 | 0.330 | 0.456 |
|  | Inverse variance weighted | 13 | 0.055 | 0.258 | 0.831 |
|  | Simple mode | 13 | -0.493 | 0.591 | 0.420 |
|  | Weighted mode | 13 | -0.481 | 0.548 | 0.397 |
| LachnospiraceaeUCG008 |  |  |  |  |  |
|  | MR Egger | 11 | -0.385 | 0.800 | 0.642 |
|  | Weighted median | 11 | -0.052 | 0.204 | 0.800 |
|  | Inverse variance weighted | 11 | 0.006 | 0.156 | 0.967 |
|  | Simple mode | 11 | -0.118 | 0.323 | 0.722 |
|  | Weighted mode | 11 | -0.107 | 0.293 | 0.723 |
| LachnospiraceaeUCG010 |  |  |  |  |  |
|  | MR Egger | 12 | 0.964 | 0.688 | 0.191 |
|  | Weighted median | 12 | 0.463 | 0.295 | 0.117 |
|  | Inverse variance weighted | 12 | 0.458 | 0.220 | 0.037 |
|  | Simple mode | 12 | 0.372 | 0.440 | 0.416 |
|  | Weighted mode | 12 | 0.380 | 0.420 | 0.385 |
| Lactobacillus |  |  |  |  |  |
|  | MR Egger | 10 | 0.718 | 0.440 | 0.141 |
|  | Weighted median | 10 | 0.091 | 0.217 | 0.677 |
|  | Inverse variance weighted | 10 | 0.059 | 0.178 | 0.739 |
|  | Simple mode | 10 | -0.079 | 0.369 | 0.835 |
|  | Weighted mode | 10 | 0.137 | 0.280 | 0.644 |
| Lactococcus |  |  |  |  |  |
|  | MR Egger | 11 | -0.831 | 0.626 | 0.218 |
|  | Weighted median | 11 | -0.009 | 0.176 | 0.957 |
|  | Inverse variance weighted | 11 | -0.044 | 0.128 | 0.728 |
|  | Simple mode | 11 | -0.075 | 0.276 | 0.793 |
|  | Weighted mode | 11 | -0.121 | 0.309 | 0.703 |
| Marvinbryantia |  |  |  |  |  |
|  | MR Egger | 12 | 0.265 | 0.810 | 0.750 |
|  | Weighted median | 12 | -0.245 | 0.280 | 0.383 |
|  | Inverse variance weighted | 12 | -0.120 | 0.211 | 0.568 |
|  | Simple mode | 12 | -0.315 | 0.434 | 0.483 |
|  | Weighted mode | 12 | -0.295 | 0.401 | 0.478 |
| Methanobrevibacter |  |  |  |  |  |
|  | MR Egger | 8 | -0.679 | 0.595 | 0.297 |
|  | Weighted median | 8 | -0.173 | 0.202 | 0.394 |
|  | Inverse variance weighted | 8 | -0.072 | 0.150 | 0.631 |
|  | Simple mode | 8 | -0.223 | 0.275 | 0.444 |
|  | Weighted mode | 8 | -0.233 | 0.262 | 0.402 |
| Odoribacter |  |  |  |  |  |
|  | MR Egger | 9 | -1.072 | 0.861 | 0.253 |
|  | Weighted median | 9 | -0.185 | 0.349 | 0.596 |
|  | Inverse variance weighted | 9 | -0.269 | 0.257 | 0.296 |
|  | Simple mode | 9 | -0.375 | 0.506 | 0.480 |
|  | Weighted mode | 9 | -0.207 | 0.519 | 0.701 |
| Olsenella |  |  |  |  |  |
|  | MR Egger | 11 | 0.122 | 0.451 | 0.792 |
|  | Weighted median | 11 | 0.070 | 0.170 | 0.679 |
|  | Inverse variance weighted | 11 | -0.008 | 0.129 | 0.951 |
|  | Simple mode | 11 | 0.296 | 0.292 | 0.336 |
|  | Weighted mode | 11 | 0.186 | 0.222 | 0.422 |
| Oscillibacter |  |  |  |  |  |
|  | MR Egger | 16 | -0.598 | 0.508 | 0.258 |
|  | Weighted median | 16 | -0.217 | 0.194 | 0.264 |
|  | Inverse variance weighted | 16 | -0.264 | 0.145 | 0.068 |
|  | Simple mode | 16 | -0.132 | 0.323 | 0.689 |
|  | Weighted mode | 16 | -0.149 | 0.306 | 0.634 |
| Oscillospira |  |  |  |  |  |
|  | MR Egger | 9 | -2.18 | 1.116 | 0.091 |
|  | Weighted median | 9 | 0.012 | 0.311 | 0.970 |
|  | Inverse variance weighted | 9 | 0.210 | 0.311 | 0.500 |
|  | Simple mode | 9 | 0.035 | 0.489 | 0.945 |
|  | Weighted mode | 9 | -0.186 | 0.420 | 0.670 |
| Oxalobacter |  |  |  |  |  |
|  | MR Egger | 12 | 0.337 | 0.605 | 0.590 |
|  | Weighted median | 12 | 0.015 | 0.172 | 0.930 |
|  | Inverse variance weighted | 12 | 0.092 | 0.132 | 0.485 |
|  | Simple mode | 12 | -0.093 | 0.242 | 0.707 |
|  | Weighted mode | 12 | -0.104 | 0.237 | 0.669 |
| Parabacteroides |  |  |  |  |  |
|  | MR Egger | 9 | -0.729 | 1.066 | 0.516 |
|  | Weighted median | 9 | -0.164 | 0.369 | 0.657 |
|  | Inverse variance weighted | 9 | 0.114 | 0.308 | 0.712 |
|  | Simple mode | 9 | -0.426 | 0.705 | 0.562 |
|  | Weighted mode | 9 | -0.474 | 0.625 | 0.471 |
| Paraprevotella |  |  |  |  |  |
|  | MR Egger | 13 | 0.072 | 0.687 | 0.918 |
|  | Weighted median | 13 | -0.071 | 0.210 | 0.735 |
|  | Inverse variance weighted | 13 | -0.010 | 0.175 | 0.953 |
|  | Simple mode | 13 | 0.024 | 0.361 | 0.949 |
|  | Weighted mode | 13 | -0.035 | 0.362 | 0.924 |
| Parasutterella |  |  |  |  |  |
|  | MR Egger | 16 | -0.010 | 0.468 | 0.983 |
|  | Weighted median | 16 | -0.077 | 0.228 | 0.737 |
|  | Inverse variance weighted | 16 | -0.124 | 0.159 | 0.435 |
|  | Simple mode | 16 | -0.488 | 0.420 | 0.264 |
|  | Weighted mode | 16 | -0.195 | 0.330 | 0.563 |
| Peptococcus |  |  |  |  |  |
|  | MR Egger | 16 | 0.043 | 0.470 | 0.928 |
|  | Weighted median | 16 | -0.128 | 0.156 | 0.409 |
|  | Inverse variance weighted | 16 | -0.247 | 0.125 | 0.048 |
|  | Simple mode | 16 | -0.120 | 0.248 | 0.633 |
|  | Weighted mode | 16 | -0.101 | 0.236 | 0.675 |
| Phascolarctobacterium |  |  |  |  |  |
|  | MR Egger | 11 | 0.394 | 0.865 | 0.659 |
|  | Weighted median | 11 | -0.222 | 0.295 | 0.452 |
|  | Inverse variance weighted | 11 | -0.254 | 0.213 | 0.232 |
|  | Simple mode | 11 | -0.296 | 0.501 | 0.568 |
|  | Weighted mode | 11 | -0.193 | 0.431 | 0.664 |
| Prevotella7 |  |  |  |  |  |
|  | MR Egger | 12 | 0.475 | 0.995 | 0.643 |
|  | Weighted median | 12 | 0.158 | 0.158 | 0.920 |
|  | Inverse variance weighted | 12 | 0.029 | 0.158 | 0.854 |
|  | Simple mode | 12 | -0.008 | 0.259 | 0.976 |
|  | Weighted mode | 12 | -0.029 | 0.239 | 0.904 |
| Prevotella9 |  |  |  |  |  |
|  | MR Egger | 18 | -0.276 | 0.398 | 0.497 |
|  | Weighted median | 18 | 0.085 | 0.203 | 0.674 |
|  | Inverse variance weighted | 18 | 0.107 | 0.143 | 0.454 |
|  | Simple mode | 18 | -0.483 | 0.367 | 0.206 |
|  | Weighted mode | 18 | 0.197 | 0.328 | 0.556 |
| RikenellaceaeRC9gutgroup |  |  |  |  |  |
|  | MR Egger | 13 | -0.103 | 0.674 | 0.881 |
|  | Weighted median | 13 | -0.061 | 0.147 | 0.675 |
|  | Inverse variance weighted | 13 | -0.070 | 0.106 | 0.509 |
|  | Simple mode | 13 | 0.248 | 0.268 | 0.372 |
|  | Weighted mode | 13 | -0.210 | 0.248 | 0.414 |
| Romboutsia |  |  |  |  |  |
|  | MR Egger | 14 | 0.368 | 0.569 | 0.530 |
|  | Weighted median | 14 | -0.158 | 0.265 | 0.550 |
|  | Inverse variance weighted | 14 | -0.020 | 0.200 | 0.921 |
|  | Simple mode | 14 | -0.316 | 0.450 | 0.494 |
|  | Weighted mode | 14 | -0.294 | 0.451 | 0.526 |
| Roseburia |  |  |  |  |  |
|  | MR Egger | 17 | 0.468 | 0.667 | 0.494 |
|  | Weighted median | 17 | -0.079 | 0.308 | 0.798 |
|  | Inverse variance weighted | 17 | -0.026 | 0.229 | 0.910 |
|  | Simple mode | 17 | -0.027 | 0.499 | 0.958 |
|  | Weighted mode | 17 | -0.182 | 0.499 | 0.721 |
| Ruminiclostridium5 |  |  |  |  |  |
|  | MR Egger | 15 | -0.239 | 0.633 | 0.712 |
|  | Weighted median | 15 | 0.018 | 0.318 | 0.954 |
|  | Inverse variance weighted | 15 | 0.005 | 0.241 | 0.984 |
|  | Simple mode | 15 | 0.299 | 0.507 | 0.564 |
|  | Weighted mode | 15 | 0.044 | 0.379 | 0.908 |
| Ruminiclostridium6 |  |  |  |  |  |
|  | MR Egger | 17 | -0.854 | 0.477 | 0.093 |
|  | Weighted median | 17 | -0.115 | 0.254 | 0.649 |
|  | Inverse variance weighted | 17 | -0.698 | 0.181 | 0.700 |
|  | Simple mode | 17 | -0.072 | 0.500 | 0.887 |
|  | Weighted mode | 17 | -0.173 | 0.444 | 0.701 |
| Ruminiclostridium9 |  |  |  |  |  |
|  | MR Egger | 15 | 1.063 | 1.205 | 0.394 |
|  | Weighted median | 15 | 0.050 | 0.305 | 0.869 |
|  | Inverse variance weighted | 15 | 0.101 | 0.242 | 0.677 |
|  | Simple mode | 15 | 0.238 | 0.487 | 0.632 |
|  | Weighted mode | 15 | 0.153 | 0.463 | 0.746 |
| RuminococcaceaeNK4A214group |  |  |  |  |  |
|  | MR Egger | 16 | -0.501 | 0.775 | 0.529 |
|  | Weighted median | 16 | -0.314 | 0.296 | 0.289 |
|  | Inverse variance weighted | 16 | -0.310 | 0.256 | 0.226 |
|  | Simple mode | 16 | 0.680 | 0.543 | 0.230 |
|  | Weighted mode | 16 | -0.593 | 0,548 | 0.296 |
| RuminococcaceaeUCG002 |  |  |  |  |  |
|  | MR Egger | 25 | 0.084 | 0.454 | 0.854 |
|  | Weighted median | 25 | -0.169 | 0.230 | 0.463 |
|  | Inverse variance weighted | 25 | -0.320 | 0.167 | 0.056 |
|  | Simple mode | 25 | 0.262 | 0.450 | 0.565 |
|  | Weighted mode | 25 | 0.060 | 0.343 | 0.862 |
| RuminococcaceaeUCG003 |  |  |  |  |  |
|  | MR Egger | 14 | 0.409 | 0.787 | 0.613 |
|  | Weighted median | 14 | 0.232 | 0.290 | 0.423 |
|  | Inverse variance weighted | 14 | 0.117 | 0.226 | 0.606 |
|  | Simple mode | 14 | 0.307 | 0.517 | 0.563 |
|  | Weighted mode | 14 | 0.272 | 0.441 | 0.547 |
| RuminococcaceaeUCG004 |  |  |  |  |  |
|  | MR Egger | 12 | 0.851 | 1.051 | 0.437 |
|  | Weighted median | 12 | -0.078 | 0.249 | 0.755 |
|  | Inverse variance weighted | 12 | 0.045 | 0.183 | 0.806 |
|  | Simple mode | 12 | -0.086 | 0.414 | 0.840 |
|  | Weighted mode | 12 | -0.101 | 0.414 | 0.813 |
| RuminococcaceaeUCG005 |  |  |  |  |  |
|  | MR Egger | 17 | 0.072 | 0.497 | 0.887 |
|  | Weighted median | 17 | 0.332 | 0.239 | 0.165 |
|  | Inverse variance weighted | 17 | 0.229 | 0.180 | 0.108 |
|  | Simple mode | 17 | 0.810 | 0.487 | 0.115 |
|  | Weighted mode | 17 | 0.667 | 0.425 | 0.136 |
| RuminococcaceaeUCG009 |  |  |  |  |  |
|  | MR Egger | 13 | -0.903 | 0.556 | 0.133 |
|  | Weighted median | 13 | 0.061 | 0.199 | 0.758 |
|  | Inverse variance weighted | 13 | 0.067 | 0.148 | 0.652 |
|  | Simple mode | 13 | 0.050 | 0.312 | 0.874 |
|  | Weighted mode | 13 | 0.038 | 0.334 | 0.911 |
| RuminococcaceaeUCG010 |  |  |  |  |  |
|  | MR Egger | 8 | 0.081 | 0.756 | 0.918 |
|  | Weighted median | 8 | -0.129 | 0.324 | 0.689 |
|  | Inverse variance weighted | 8 | -0.277 | 0.246 | 0.259 |
|  | Simple mode | 8 | -0.185 | 0.514 | 0.729 |
|  | Weighted mode | 8 | -0.127 | 0.421 | 0.771 |
| RuminococcaceaeUCG011 |  |  |  |  |  |
|  | MR Egger | 8 | -0.902 | 0.806 | 0.306 |
|  | Weighted median | 8 | 0.000 | 0.182 | 0.998 |
|  | Inverse variance weighted | 8 | -0.066 | 0.162 | 0.683 |
|  | Simple mode | 8 | 0.595 | 0.325 | 0.860 |
|  | Weighted mode | 8 | -0.394 | 0.301 | 0.232 |
| RuminococcaceaeUCG013 |  |  |  |  |  |
|  | MR Egger | 14 | -0.147 | 0.664 | 0.828 |
|  | Weighted median | 14 | 0.125 | 0.308 | 0.684 |
|  | Inverse variance weighted | 14 | 0.949 | 0.220 | 0.666 |
|  | Simple mode | 14 | 0.688 | 0.546 | 0.229 |
|  | Weighted mode | 14 | 0.357 | 0.466 | 0.458 |
| RuminococcaceaeUCG014 |  |  |  |  |  |
|  | MR Egger | 17 | 0.435 | 0.444 | 0.342 |
|  | Weighted median | 17 | 0.242 | 0.242 | 0.061 |
|  | Inverse variance weighted | 17 | 0.171 | 0.171 | 0.050 |
|  | Simple mode | 17 | 0.374 | 0.374 | 0.205 |
|  | Weighted mode | 17 | 0.305 | 0.305 | 0.125 |
| Ruminococcus1 |  |  |  |  |  |
|  | MR Egger | 14 | -1.112 | 0.592 | 0.085 |
|  | Weighted median | 14 | -0.727 | 0.279 | 0.009 |
|  | Inverse variance weighted | 14 | -0.402 | 0.212 | 0.058 |
|  | Simple mode | 14 | -0.935 | 0.501 | 0.085 |
|  | Weighted mode | 14 | -0.904 | 0.470 | 0.077 |
| Ruminococcus2 |  |  |  |  |  |
|  | MR Egger | 15 | 0.533 | 0.435 | 0.242 |
|  | Weighted median | 15 | 0.233 | 0.234 | 0.318 |
|  | Inverse variance weighted | 15 | 0.207 | 0.181 | 0.251 |
|  | Simple mode | 15 | 0.298 | 0.365 | 0.428 |
|  | Weighted mode | 15 | 0.424 | 0.306 | 0.187 |
| Sellimonas |  |  |  |  |  |
|  | MR Egger | 11 | -0.201 | 0.767 | 0.799 |
|  | Weighted median | 11 | -0.103 | 0.156 | 0.512 |
|  | Inverse variance weighted | 11 | -0.153 | 0.117 | 0.192 |
|  | Simple mode | 11 | -0.203 | 0.278 | 0.482 |
|  | Weighted mode | 11 | 0.139 | 0.275 | 0.623 |
| Senegalimassilia |  |  |  |  |  |
|  | MR Egger | 8 | -1.165 | 0.985 | 0.282 |
|  | Weighted median | 8 | 0.016 | 0.270 | 0.953 |
|  | Inverse variance weighted | 8 | 0.001 | 0.201 | 0.995 |
|  | Simple mode | 8 | 0.082 | 0.402 | 0.843 |
|  | Weighted mode | 8 | 0.089 | 0.397 | 0.829 |
| Slackia |  |  |  |  |  |
|  | MR Egger | 9 | -0.506 | 1.160 | 0.676 |
|  | Weighted median | 9 | 0.344 | 0.257 | 0.181 |
|  | Inverse variance weighted | 9 | 0.114 | 0.238 | 0.632 |
|  | Simple mode | 9 | 0.593 | 0.468 | 0.241 |
|  | Weighted mode | 9 | 0.568 | 0.452 | 0.244 |
| Streptococcus |  |  |  |  |  |
|  | MR Egger | 17 | -0.890 | 0.782 | 0.273 |
|  | Weighted median | 17 | -0.124 | 0.280 | 0.657 |
|  | Inverse variance weighted | 17 | -0.373 | 0.202 | 0.064 |
|  | Simple mode | 17 | 0.049 | 0.467 | 0.917 |
|  | Weighted mode | 17 | 0.080 | 0.510 | 0.877 |
| Subdoligranulum |  |  |  |  |  |
|  | MR Egger | 14 | 0.231 | 0.589 | 0.702 |
|  | Weighted median | 14 | -0.048 | 0.302 | 0.874 |
|  | Inverse variance weighted | 14 | -0.080 | 0.213 | 0.706 |
|  | Simple mode | 14 | -0.136 | 0.453 | 0.769 |
|  | Weighted mode | 14 | -0.016 | 0.408 | 0.969 |
| Sutterella |  |  |  |  |  |
|  | MR Egger | 12 | -0.581 | 1.336 | 0.673 |
|  | Weighted median | 12 | -0.067 | 0.329 | 0.839 |
|  | Inverse variance weighted | 12 | -0.001 | 0.296 | 0.998 |
|  | Simple mode | 12 | -0.041 | 0.555 | 0.943 |
|  | Weighted mode | 12 | -0.067 | 0.481 | 0.891 |
| Terrisporobacter |  |  |  |  |  |
|  | MR Egger | 6 | 0.601 | 0.730 | 0.457 |
|  | Weighted median | 6 | -0.254 | 0.283 | 0.371 |
|  | Inverse variance weighted | 6 | -0.249 | 0.225 | 0.268 |
|  | Simple mode | 6 | -0.206 | 0.442 | 0.660 |
|  | Weighted mode | 6 | 0.017 | 0.434 | 0.970 |
| Turicibacter |  |  |  |  |  |
|  | MR Egger | 14 | 0.083 | 0.809 | 0.920 |
|  | Weighted median | 14 | -0.222 | 0.229 | 0.332 |
|  | Inverse variance weighted | 14 | -0.182 | 0.190 | 0.338 |
|  | Simple mode | 14 | -0.073 | 0.407 | 0.860 |
|  | Weighted mode | 14 | -0.217 | 0.394 | 0.592 |
| Tyzzerella3 |  |  |  |  |  |
|  | MR Egger | 13 | -0.601 | 0.782 | 0.457 |
|  | Weighted median | 13 | -0.092 | 0.182 | 0.614 |
|  | Inverse variance weighted | 13 | -0.085 | 0.131 | 0.519 |
|  | Simple mode | 13 | -0.278 | 0.326 | 0.410 |
|  | Weighted mode | 13 | -0.170 | 0.314 | 0.597 |
| Veillonella |  |  |  |  |  |
|  | MR Egger | 8 | -4.24 | 2.087 | 0.088 |
|  | Weighted median | 8 | -0.26 | 0.298 | 0.382 |
|  | Inverse variance weighted | 8 | -0.42 | 0.223 | 0.061 |
|  | Simple mode | 8 | 0.260 | 0.423 | 0.558 |
|  | Weighted mode | 8 | -0.254 | 0.383 | 0.528 |
| Victivallis |  |  |  |  |  |
|  | MR Egger | 12 | 0.565 | 0.737 | 0.461 |
|  | Weighted median | 12 | 0.125 | 0.158 | 0.430 |
|  | Inverse variance weighted | 12 | 0.138 | 0.111 | 0.214 |
|  | Simple mode | 12 | 0.124 | 0.255 | 0.635 |
|  | Weighted mode | 12 | 0.111 | 0.254 | 0.671 |
| Order |  |  |  |  |  |
| Actinomycetales |  |  |  |  |  |
|  | MR Egger | 4 | 0.785 | 0.573 | 0.304 |
|  | Weighted median | 4 | -0.118 | 0.316 | 0.708 |
|  | Inverse variance weighted | 4 | -0.000 | 0.262 | 0.999 |
|  | Simple mode | 4 | -0.408 | 0.553 | 0.514 |
|  | Weighted mode | 4 | 0.394 | 0.366 | 0.360 |
| Bacillales |  |  |  |  |  |
|  | MR Egger | 9 | 0.254 | 0.636 | 0.701 |
|  | Weighted median | 9 | 0.226 | 0.170 | 0.183 |
|  | Inverse variance weighted | 9 | 0.057 | 0.144 | 0.694 |
|  | Simple mode | 9 | 0.260 | 0.257 | 0.341 |
|  | Weighted mode | 9 | 0.256 | 0.226 | 0.290 |
| Bacteroidales |  |  |  |  |  |
|  | MR Egger | 14 | 0.490 | 0.585 | 0.418 |
|  | Weighted median | 14 | 0.320 | 0.319 | 0.316 |
|  | Inverse variance weighted | 14 | 0.428 | 0.232 | 0.065 |
|  | Simple mode | 14 | -0.043 | 0.545 | 0.939 |
|  | Weighted mode | 14 | -0.024 | 0.517 | 0.963 |
| Bifidobacteriales |  |  |  |  |  |
|  | MR Egger | 17 | -0.405 | 0.644 | 0.539 |
|  | Weighted median | 17 | -0.033 | 0.247 | 0.894 |
|  | Inverse variance weighted | 17 | 0.082 | 0.175 | 0.639 |
|  | Simple mode | 17 | -0.268 | 0.498 | 0.598 |
|  | Weighted mode | 17 | -0.310 | 0.354 | 0.394 |
| Burkholderiales |  |  |  |  |  |
|  | MR Egger | 12 | -0.006 | 0.716 | 0.993 |
|  | Weighted median | 12 | 0.052 | 0.308 | 0.865 |
|  | Inverse variance weighted | 12 | 0.142 | 0.234 | 0.544 |
|  | Simple mode | 12 | 0.108 | 0.434 | 0.809 |
|  | Weighted mode | 12 | 0.131 | 0.438 | 0.771 |
| Clostridiales |  |  |  |  |  |
|  | MR Egger | 15 | -0.670 | 0.901 | 0.470 |
|  | Weighted median | 15 | -0.327 | 0.294 | 0.266 |
|  | Inverse variance weighted | 15 | -0.214 | 0.220 | 0.331 |
|  | Simple mode | 15 | -0.499 | 0.472 | 0.308 |
|  | Weighted mode | 15 | -0.415 | 0.403 | 0.320 |
| Coriobacteriales |  |  |  |  |  |
|  | MR Egger | 17 | 0.410 | 0.485 | 0.411 |
|  | Weighted median | 17 | 0.346 | 0.262 | 0.187 |
|  | Inverse variance weighted | 17 | 0.300 | 0.193 | 0.121 |
|  | Simple mode | 17 | 0.302 | 0.450 | 0.513 |
|  | Weighted mode | 17 | 0.375 | 0.360 | 0.313 |
| Desulfovibrionales |  |  |  |  |  |
|  | MR Egger | 13 | 0.042 | 0.543 | 0.940 |
|  | Weighted median | 13 | -0.138 | 0.300 | 0.646 |
|  | Inverse variance weighted | 13 | -0.233 | 0.206 | 0.258 |
|  | Simple mode | 13 | -0.200 | 0.442 | 0.660 |
|  | Weighted mode | 13 | -0.141 | 0.362 | 0.704 |
| Enterobacteriales |  |  |  |  |  |
|  | MR Egger | 11 | 0.637 | 1.098 | 0.576 |
|  | Weighted median | 11 | -0.022 | 0.330 | 0.948 |
|  | Inverse variance weighted | 11 | 0.290 | 0.240 | 0.226 |
|  | Simple mode | 11 | -0.181 | 0.503 | 0.727 |
|  | Weighted mode | 11 | -0.190 | 0.494 | 0.708 |
| Erysipelotrichales |  |  |  |  |  |
|  | MR Egger | 13 | -0.700 | 1.317 | 0.608 |
|  | Weighted median | 13 | -0.215 | 0.374 | 0.565 |
|  | Inverse variance weighted | 13 | -0.111 | 0.294 | 0.706 |
|  | Simple mode | 13 | -0.533 | 0.662 | 0.437 |
|  | Weighted mode | 13 | -0.560 | 0.611 | 0.378 |
| Gastranaerophilales |  |  |  |  |  |
|  | MR Egger | 12 | -0.631 | 0.462 | 0.202 |
|  | Weighted median | 12 | 0.176 | 0.210 | 0.402 |
|  | Inverse variance weighted | 12 | 0.009 | 0.152 | 0.950 |
|  | Simple mode | 12 | 0.368 | 0.377 | 0.350 |
|  | Weighted mode | 12 | 0.305 | 0.360 | 0.414 |
| Lactobacillales |  |  |  |  |  |
|  | MR Egger | 19 | 0.196 | 0.451 | 0.670 |
|  | Weighted median | 19 | -0.153 | 0.259 | 0.554 |
|  | Inverse variance weighted | 19 | -0.182 | 0.183 | 0.319 |
|  | Simple mode | 19 | -0.494 | 0.491 | 0.328 |
|  | Weighted mode | 19 | 0.155 | 0.429 | 0.721 |
| Methanobacteriales |  |  |  |  |  |
|  | MR Egger | 12 | -0.990 | 0.457 | 0.056 |
|  | Weighted median | 12 | -0.171 | 0.164 | 0.296 |
|  | Inverse variance weighted | 12 | -0.029 | 0.126 | 0.818 |
|  | Simple mode | 12 | -0.251 | 0.274 | 0.378 |
|  | Weighted mode | 12 | -0.257 | 0.243 | 0.314 |
| MollicutesRF9 |  |  |  |  |  |
|  | MR Egger | 16 | 0.531 | 0.631 | 0.415 |
|  | Weighted median | 16 | -0.040 | 0.247 | 0.870 |
|  | Inverse variance weighted | 16 | 0.021 | 0.199 | 0.916 |
|  | Simple mode | 16 | -0.552 | 0.568 | 0.346 |
|  | Weighted mode | 16 | -0.480 | 0.579 | 0.420 |
| NB1n |  |  |  |  |  |
|  | MR Egger | 15 | -0.077 | 0.521 | 0.884 |
|  | Weighted median | 15 | -0.201 | 0.158 | 0.203 |
|  | Inverse variance weighted | 15 | -0.227 | 0.118 | 0.054 |
|  | Simple mode | 15 | -0.294 | 0.259 | 0.275 |
|  | Weighted mode | 15 | -0.289 | 0.245 | 0.259 |
| Pasteurellales |  |  |  |  |  |
|  | MR Egger | 19 | -0.129 | 0.306 | 0.677 |
|  | Weighted median | 19 | 0.075 | 0.183 | 0.680 |
|  | Inverse variance weighted | 19 | 0.145 | 0.132 | 0.270 |
|  | Simple mode | 19 | 0.165 | 0.289 | 0.575 |
|  | Weighted mode | 19 | 0.051 | 0.269 | 0.852 |
| Rhodospirillales |  |  |  |  |  |
|  | MR Egger | 15 | -0.938 | 0.593 | 0.138 |
|  | Weighted median | 15 | 0.047 | 0.217 | 0.827 |
|  | Inverse variance weighted | 15 | -0.066 | 0.148 | 0.656 |
|  | Simple mode | 15 | 0.134 | 0.396 | 0.741 |
|  | Weighted mode | 15 | 0.151 | 0.418 | 0.723 |
| Selenomonadales |  |  |  |  |  |
|  | MR Egger | 13 | -1.162 | 1.158 | 0.337 |
|  | Weighted median | 13 | 0.022 | 0.379 | 0.954 |
|  | Inverse variance weighted | 13 | -0.180 | 0.327 | 0.582 |
|  | Simple mode | 13 | -0.975 | 0.671 | 0.172 |
|  | Weighted mode | 13 | 0.121 | 0.649 | 0.856 |
| Verrucomicrobiales |  |  |  |  |  |
|  | MR Egger | 12 | -0.113 | 0.702 | 0.875 |
|  | Weighted median | 12 | 0.105 | 0.268 | 0.697 |
|  | Inverse variance weighted | 12 | 0.258 | 0.199 | 0.194 |
|  | Simple mode | 12 | -0.027 | 0.412 | 0.950 |
|  | Weighted mode | 12 | -0.054 | 0.397 | 0.893 |
| Victivallales |  |  |  |  |  |
|  | MR Egger | 10 | 0.221 | 0.617 | 0.729 |
|  | Weighted median | 10 | 0.054 | 0.195 | 0.783 |
|  | Inverse variance weighted | 10 | 0.206 | 0.154 | 0.180 |
|  | Simple mode | 10 | 0.008 | 0.324 | 0.981 |
|  | Weighted mode | 10 | 0.002 | 0.299 | 0.995 |
| Phylum |  |  |  |  |  |
| Actinobacteria |  |  |  |  |  |
|  | MR Egger | 18 | 0.004 | 0.914 | 0.997 |
|  | Weighted median | 18 | 0.320 | 0.277 | 0.248 |
|  | Inverse variance weighted | 18 | 0.272 | 0.199 | 0.171 |
|  | Simple mode | 18 | 0.396 | 0.504 | 0.443 |
|  | Weighted mode | 18 | 0.387 | 0.448 | 0.400 |
| Bacteroidetes |  |  |  |  |  |
|  | MR Egger | 12 | 0.679 | 0.587 | 0.274 |
|  | Weighted median | 12 | 0.118 | 0.341 | 0.729 |
|  | Inverse variance weighted | 12 | 0.356 | 0.244 | 0.145 |
|  | Simple mode | 12 | -0.181 | 0.558 | 0.752 |
|  | Weighted mode | 12 | -0.152 | 0.508 | 0.771 |
| Cyanobacteria |  |  |  |  |  |
|  | MR Egger | 10 | -0.047 | 0.770 | 0.953 |
|  | Weighted median | 10 | 0.098 | 0.244 | 0.688 |
|  | Inverse variance weighted | 10 | -0.033 | 0.224 | 0.882 |
|  | Simple mode | 10 | 0.352 | 0.389 | 0.389 |
|  | Weighted mode | 10 | 0.215 | 0.366 | 0.572 |
| Euryarchaeota |  |  |  |  |  |
|  | MR Egger | 13 | 0.221 | 0.497 | 0.666 |
|  | Weighted median | 13 | 0.072 | 0.154 | 0.642 |
|  | Inverse variance weighted | 13 | 0.099 | 0.113 | 0.380 |
|  | Simple mode | 13 | 0.079 | 0.272 | 0.777 |
|  | Weighted mode | 13 | 0.042 | 0.261 | 0.874 |
| Firmicutes |  |  |  |  |  |
|  | MR Egger | 19 | -0.162 | 0.575 | 0.782 |
|  | Weighted median | 19 | -0.104 | 0.283 | 0.715 |
|  | Inverse variance weighted | 19 | 0.140 | 0.201 | 0.487 |
|  | Simple mode | 19 | -0.327 | 0.488 | 0.511 |
|  | Weighted mode | 19 | -0.193 | 0.476 | 0.690 |
| Lentisphaerae |  |  |  |  |  |
|  | MR Egger | 11 | 0.109 | 0.595 | 0.859 |
|  | Weighted median | 11 | 0.034 | 0.190 | 0.857 |
|  | Inverse variance weighted | 11 | 0.170 | 0.142 | 0.230 |
|  | Simple mode | 11 | -0.030 | 0.303 | 0.922 |
|  | Weighted mode | 11 | -0.042 | 0.277 | 0.881 |
| Proteobacteria |  |  |  |  |  |
|  | MR Egger | 14 | 0.561 | 0.628 | 0.390 |
|  | Weighted median | 14 | -0.299 | 0.302 | 0.323 |
|  | Inverse variance weighted | 14 | -0.370 | 0.220 | 0.092 |
|  | Simple mode | 14 | -0.435 | 0.501 | 0.401 |
|  | Weighted mode | 14 | 0.063 | 0.433 | 0.886 |
| Tenericutes |  |  |  |  |  |
|  | MR Egger | 12 | 1.080 | 0.774 | 0.193 |
|  | Weighted median | 12 | -0.125 | 0.296 | 0.672 |
|  | Inverse variance weighted | 12 | -0.077 | 0.244 | 0.751 |
|  | Simple mode | 12 | -0.834 | 0.599 | 0.191 |
|  | Weighted mode | 12 | -0.718 | 0.573 | 0.236 |
| Verrucomicrobia |  |  |  |  |  |
|  | MR Egger | 12 | -0.187 | 0.516 | 0.724 |
|  | Weighted median | 12 | 0.016 | 0.268 | 0.951 |
|  | Inverse variance weighted | 12 | 0.120 | 0.196 | 0.540 |
|  | Simple mode | 12 | -0.037 | 0.470 | 0.939 |
|  | Weighted mode | 12 | -0.152 | 0.365 | 0.685 |

**Table 2** Characteristics of the genetic variants associated with 9 bacterial that have been identified to be associated with the risk of Hyperprolactinemia.

| **Gut microbiota** | **SNP** | **Effect allele** | **Beta** | **SE** | ***p*-value** |
| --- | --- | --- | --- | --- | --- |
| Family Acidaminococcaceae | rs2933324 | A | -0.066255706 | 0.014025652 | 2.24E-06 |
|  | rs6923842 | T | -0.079610621 | 0.016919247 | 2.21E-06 |
|  | rs6589457 | A | 0.165910976 | 0.034951601 | 2.32E-06 |
|  | rs6427992 | G | -0.059585662 | 0.012960033 | 4.24E-06 |
|  | rs262812 | T | -0.065674997 | 0.014225656 | 3.25E-06 |
|  | rs74540770 | G | -0.108969098 | 0.024353266 | 7.09E-06 |
|  | rs45497800 | T | -0.117862982 | 0.025735121 | 5.86E-06 |
|  | rs78702810 | T | -0.143755204 | 0.032324372 | 9.16E-06 |
| Genus Eubacteriumruminantiumgroup | rs2116427 | A | 0.091146051 | 0.018235307 | 4.67E-07 |
|  | rs139749 | C | -0.084543927 | 0.017179078 | 8.59E-07 |
|  | rs72836424 | C | -0.139824589 | 0.030068963 | 2.62E-06 |
|  | rs2229917 | A | 0.153537929 | 0.032392215 | 2.16E-06 |
|  | rs10131724 | A | -0.199832279 | 0.041457713 | 2.39E-06 |
|  | rs16891896 | G | -0.174787011 | 0.039057268 | 2.38E-06 |
|  | rs7000472 | A | -0.076228208 | 0.016523022 | 4.07E-06 |
|  | rs2418654 | C | -0.074887891 | 0.016585161 | 6.17E-06 |
|  | rs13025464 | T | -0.073707715 | 0.016378577 | 6.97E-06 |
|  | rs6676699 | G | -0.088812364 | 0.019644717 | 6.38E-06 |
|  | rs73139629 | A | -0.115097848 | 0.024790941 | 5.36E-06 |
|  | rs112375806 | T | 0.143141085 | 0.029375749 | 5.82E-06 |
|  | rs606117 | A | 0.083324266 | 0.018055987 | 4.82E-06 |
|  | rs57340348 | T | -0.097942933 | 0.021216595 | 4.93E-06 |
|  | rs2817174 | C | -0.073430579 | 0.016368685 | 7.87E-06 |
|  | rs10923018 | G | 0.072643786 | 0.016092406 | 6.80E-06 |
|  | rs11637981 | G | -0.073257996 | 0.016088796 | 5.44E-06 |
|  | rs17519472 | C | 0.10780414 | 0.023398378 | 4.70E-06 |
|  | rs209813 | G | -0.103488116 | 0.023639057 | 9.23E-06 |
| Genus Ruminococcusgauvreauiigroup | rs2047242 | A | -0.06760301 | 0.013373773 | 2.46E-07 |
|  | rs1391597 | C | 0.059028381 | 0.012487351 | 1.86E-06 |
|  | rs71386687 | T | 0.121036908 | 0.02385996 | 2.91E-07 |
|  | rs9870933 | A | 0.062164279 | 0.012607163 | 8.49E-07 |
|  | rs431418 | A | -0.094737043 | 0.02101571 | 5.54E-06 |
|  | rs10931481 | G | 0.061009638 | 0.013048446 | 3.38E-06 |
|  | rs289410 | G | -0.065492577 | 0.013910104 | 2.27E-06 |
|  | rs2105937 | A | 0.058021543 | 0.012780587 | 5.10E-06 |
|  | rs2166943 | A | 0.056697379 | 0.012349673 | 5.28E-06 |
|  | rs12539819 | C | 0.110653536 | 0.02406836 | 4.49E-06 |
|  | rs12079579 | A | 0.095532379 | 0.021343619 | 5.04E-06 |
|  | rs73802842 | C | 0.07368081 | 0.016966478 | 7.48E-06 |
|  | rs13188803 | T | 0.070941873 | 0.015685519 | 7.28E-06 |
| Genus Anaerofilum | rs712981 | A | 0.100759617 | 0.020290248 | 6.83E-07 |
|  | rs816292 | T | -0.113002639 | 0.022040046 | 2.64E-07 |
|  | rs79598899 | C | 0.182585804 | 0.035728447 | 3.75E-07 |
|  | rs4506496 | G | 0.103089882 | 0.021286122 | 1.49E-06 |
|  | rs17105491 | G | -0.19310744 | 0.04111866 | 1.57E-06 |
|  | rs10794359 | T | -0.095352431 | 0.020059979 | 2.23E-06 |
|  | rs17096874 | C | -0.126336895 | 0.02689189 | 2.86E-06 |
|  | rs356049 | G | 0.132602907 | 0.028980209 | 6.56E-06 |
|  | rs1563175 | A | 0.092383381 | 0.020215708 | 5.54E-06 |
|  | rs17012738 | T | 0.090341716 | 0.020023943 | 7.24E-06 |
|  | rs9299345 | T | -0.136399548 | 0.030237163 | 8.04E-06 |
|  | rs4244069 | G | -0.146774518 | 0.032659424 | 9.81E-06 |
| Genus Eisenbergiella | rs3812426 | G | 0.106447316 | 0.0224159 | 2.72E-06 |
|  | rs1508033 | A | 0.091545961 | 0.019578652 | 3.23E-06 |
|  | rs12278566 | T | -0.121064706 | 0.025165804 | 1.65E-06 |
|  | rs2683098 | C | 0.107318939 | 0.022515029 | 2.24E-06 |
|  | rs11079158 | T | 0.100626785 | 0.022546032 | 7.35E-06 |
|  | rs12257723 | A | -0.095274183 | 0.021156892 | 8.85E-06 |
|  | rs13258851 | A | 0.137001443 | 0.030212434 | 7.75E-06 |
|  | rs12710729 | C | 0.089338392 | 0.019906928 | 9.84E-06 |
|  | rs4462860 | G | 0.093907092 | 0.020110429 | 4.16E-06 |
|  | rs11027642 | C | 0.129005538 | 0.028484392 | 4.92E-06 |
|  | rs11938607 | T | 0.097812525 | 0.021663562 | 8.22E-06 |
|  | rs1553971 | T | 0.120964524 | 0.026305423 | 5.27E-06 |
|  | rs3812426 | G | 0.106447316 | 0.0224159 | 2.72E-06 |
| Genus ErysipelotrichaceaeUCG003 | rs76502207 | T | 0.14484376 | 0.028992913 | 6.41E-07 |
|  | rs28568391 | A | -0.058391081 | 0.011874052 | 6.42E-07 |
|  | rs11994308 | C | 0.115417413 | 0.024250252 | 1.33E-06 |
|  | rs10164067 | T | -0.103260131 | 0.021240502 | 1.13E-06 |
|  | rs8053479 | A | -0.083821185 | 0.018650912 | 5.83E-06 |
|  | rs62403464 | T | -0.073174326 | 0.015651572 | 3.44E-06 |
|  | rs17798136 | G | 0.15881755 | 0.034767431 | 3.24E-06 |
|  | rs59104037 | A | -0.095126261 | 0.020474209 | 4.48E-06 |
|  | rs11666127 | A | -0.07191871 | 0.016137058 | 7.90E-06 |
|  | rs59068084 | T | 0.056477243 | 0.012022387 | 3.12E-06 |
|  | rs73074432 | C | 0.072153999 | 0.016437785 | 9.99E-06 |
|  | rs4758231 | G | -0.05517544 | 0.012204675 | 6.55E-06 |
|  | rs74988980 | G | -0.133141406 | 0.034857439 | 8.64E-06 |
|  | rs75949021 | T | -0.16966706 | 0.037423793 | 3.58E-06 |
|  | rs6875357 | C | 0.165673441 | 0.035383116 | 6.70E-06 |
|  | rs79396538 | C | 0.084814752 | 0.019114638 | 8.63E-06 |
|  | rs12251396 | A | -0.070539949 | 0.015876598 | 9.52E-06 |
| Genus RuminococcaceaeUCG014 | rs72809222 | T | 0.067177506 | 0.013983796 | 2.41E-06 |
|  | rs115777838 | T | -0.188349525 | 0.038664295 | 4.62E-07 |
|  | rs12638134 | T | 0.058254795 | 0.011965732 | 1.21E-06 |
|  | rs56105232 | G | 0.139275689 | 0.029913177 | 2.91E-06 |
|  | rs995642 | C | 0.060047967 | 0.012641687 | 1.90E-06 |
|  | rs10941294 | C | -0.122057405 | 0.026001669 | 2.40E-06 |
|  | rs79640386 | T | -0.110863476 | 0.024809787 | 8.74E-06 |
|  | rs439810 | G | -0.057712693 | 0.012667538 | 7.04E-06 |
|  | rs10495392 | C | -0.082485954 | 0.018719441 | 9.96E-06 |
|  | rs62478832 | T | -0.058128986 | 0.012903031 | 6.04E-06 |
|  | rs61898819 | A | 0.060786464 | 0.01387607 | 9.92E-06 |
|  | rs853612 | A | -0.05282572 | 0.011936668 | 9.75E-06 |
|  | rs73186226 | G | -0.099385194 | 0.02168186 | 6.72E-06 |
|  | rs34402072 | C | -0.068802318 | 0.015608285 | 9.80E-06 |
|  | rs74060145 | C | -0.115766761 | 0.025419855 | 8.71E-06 |
|  | rs77627087 | C | 0.067561994 | 0.015010339 | 7.43E-06 |
|  | rs17296933 | C | -0.08295439 | 0.018505763 | 7.34E-06 |
|  | rs10791168 | A | -0.066462357 | 0.015006876 | 9.76E-06 |
| Family BacteroidalesS24.7group | rs738193 | T | 0.084732776 | 0.016585588 | 3.82E-07 |
|  | rs941000 | C | 0.085026574 | 0.016338237 | 3.16E-07 |
|  | rs689695 | C | 0.081457787 | 0.016705675 | 1.28E-06 |
|  | rs17043785 | T | -0.176186835 | 0.034713295 | 5.12E-07 |
|  | rs10872669 | A | -0.123070536 | 0.027566518 | 9.49E-06 |
|  | rs12748533 | G | -0.082110804 | 0.017270056 | 2.59E-06 |
|  | rs6831034 | T | -0.095654602 | 0.020522806 | 6.10E-06 |
|  | rs61508842 | T | 0.122574311 | 0.027238851 | 7.83E-06 |
|  | rs78609301 | A | -0.086706514 | 0.019570857 | 7.09E-06 |
|  | rs7217209 | C | 0.084301877 | 0.018730791 | 8.43E-06 |
|  | rs11135366 | C | 0.084214168 | 0.01837388 | 8.78E-06 |
| genus.Peptococcus.id.2037 | rs10031059 | T | -0.121166531 | 0.022584403 | 1.24E-07 |
|  | rs75754569 | C | 0.181434144 | 0.031942874 | 1.10E-08 |
|  | rs77681628 | C | 0.200307214 | 0.038732756 | 2.69E-07 |
|  | rs62424012 | G | 0.137163822 | 0.029213555 | 1.15E-06 |
|  | rs413827 | G | 0.11022947 | 0.023752348 | 3.30E-06 |
|  | rs2054133 | G | 0.089543257 | 0.018833153 | 2.14E-06 |
|  | rs11001941 | G | -0.195611127 | 0.039221681 | 1.33E-06 |
|  | rs7033353 | T | 0.090152001 | 0.018995009 | 2.22E-06 |
|  | rs5770862 | T | 0.162017845 | 0.035681339 | 3.22E-06 |
|  | rs34282744 | G | 0.19179739 | 0.03998324 | 1.84E-06 |
|  | rs72850165 | T | -0.134304018 | 0.030042318 | 5.74E-06 |
|  | rs11030569 | A | -0.174020126 | 0.037413336 | 3.13E-06 |
|  | rs7766680 | G | 0.09768317 | 0.021415492 | 3.51E-06 |
|  | rs74592222 | G | 0.137957011 | 0.030296488 | 8.55E-06 |
|  | rs36121075 | A | -0.140670082 | 0.030627993 | 6.99E-06 |
|  | rs12069354 | C | 0.167629782 | 0.037950049 | 9.28E-06 |

**Supplementary Table 3** Details of the genetic variants with potential pleiotropy among instrumental variables used for gut microbiota

| **Gut microbiotia** | **SNP** | **Pleitropic trait** | ***p*-value** | **PMID** |
| --- | --- | --- | --- | --- |
| family.Acidaminococcaceae | rs45497800 | Systemic lupus erythematosus | 1.96E-08 | 18204098 |
|  |  | Anti dsDNA autoantibody status in systemic lupus erythematosus SLE patients | 8.1E-10 | 21408207 |
|  |  | Atopic dermatitis | 8.83E-13 | 23042114 |
|  |  | Systemic lupus erythematosus SLE | 1.74E-08 | 23053960 |
|  |  | Rheumatoid arthritis | 1.4E-06 | 24390342 |
| family.BacteroidalesS24.7group | rs78609301 | Sum eosinophil basophil counts | 3.27E-18 | 27863252 |
| genus.Ruminococcusgauvreauiigroup | rs71386687 | Basophil count | 5.99E-16 | 27863252 |
|  |  | Basophil percentage of white cells | 4.28E-14 | 27863252 |
| genus.Ruminococcusgauvreauiigroup | rs9870933 | Eosinophil count | 8.48E-13 | 27863252 |
| genus.Ruminococcusgauvreauiigroup | rs10931481 | Neutrophil percentage of granulocytes | 8.68E-13 | 27863252 |
|  |  | Basophil percentage of granulocytes | 1.74E-12 | 27863252 |
|  |  | Eosinophil percentage of white cells | 2.73E-10 | 27863252 |
|  |  | Eosinophil percentage of granulocytes | 2.75E-08 | 27863252 |
|  |  | Mean corpuscular volume | 1.76E-19 | 27863252 |
|  |  | Mean corpuscular hemoglobin | 6.37E-14 | 27863252 |
|  |  | Neutrophil count | 3.58E-06 | 27863252 |
| genus.Eisenbergiella | rs12278566 | Sum basophil neutrophil counts | 4.14E-06 | 27863252 |
| genus.Eisenbergiella | rs12257723 | Myeloid white cell count | 5.51E-06 | 27863252 |
| genus.ErysipelotrichaceaeUCG003 | rs17798136 | Sum neutrophil eosinophil counts | 7.86E-06 | 27863252 |
| genus.ErysipelotrichaceaeUCG003 | rs59104037 | Granulocyte count | 8.67E-06 | 27863252 |
